# Glycemia reprograms substrate transport and shapes divergent stress responses in differentiating H9c2 cells

**DOI:** 10.15698/cst2026.10.321

**Published:** 2026-10-05

**Authors:** Sofia Passaponti, Asia Iserani, Roberta Romagnoli, Laura Cresti, Cinzia Del Gaudio, Alice Luddi, Leonardo Ermini, Francesca Ietta

**Affiliations:** 1 Department of Medicine, Surgery and Neuroscience, University of Siena, Siena, Italy; 2 Department of Life Sciences, University of Siena, Siena, Italy; 3 Department of Molecular and Developmental Medicine, University of Siena, Siena, Italy

**Keywords:** glycemic stress, cardiac metabolism, fetal development, maternal malnutrition, pregnancy

## Abstract

Metabolic stress is a major determinant of cell fate, particularly during differentiation, when long-lasting adaptations shape cellular responses to subsequent insults. Early-life glycemic instability is increasingly recognized as a stressor capable of programming cardiomyocyte metabolism and vulnerability, yet its direct impact on stress-response pathways during cardiomyocyte differentiation remains poorly defined. Here, we used differentiating H9c2 cardiomyoblasts to model chronic hypoglycemic (2.5 mM), normoglycemic (5 mM), and hyperglycemic (30 mM) conditions as opposing metabolic challenges, and examined their effects on substrate transport, lipid homeostasis, and stress-induced cell death pathways.

Both hypoglycemic and hyperglycemic stress reprogrammed phenotypes, but through distinct mechanisms. Hypoglycemic stress increased expression of the glucose transporter GLUT1, concomitantly enhancing functional glucose uptake. It also upregulated the fatty-acid transporter CD36 and carnitine palmitoyltransferase-1 (CPT1), indicating coordinated enhancement of substrate uptake and mitochondrial fatty-acid oxidation. In contrast, hyperglycemic stress suppressed GLUT1 expression and decreased glucose uptake, while increasing CD36 without upregulating CPT1. Under iso-osmotic conditions, this resulted in intracellular lipid accumulation consistent with an overload and lipotoxic stress. When challenged with anoxia–reoxygenation, hypoglycemia-conditioned cells predominantly engaged autophagy, as shown by increased LC3B-II/LC3B-I ratio, whereas hyperglycemia-conditioned cells exhibited enhanced apoptotic signaling, with increased caspase-3, PARP cleavage and DNA fragmentation. Notably, normoglycemic differentiation conferred the greatest resistance to oxygen fluctuations.

Together, these findings demonstrate that chronic glycemic stress during cardiomyoblast differentiation programs divergent stress-response pathways, biasing cells toward autophagy or apoptosis in response to acute injury. This work highlights metabolic homeostasis as a critical determinant of cellular stress resilience and provides mechanistic insight into how early metabolic stress shapes cardiomyocyte vulnerability.

## INTRODUCTION

Early developmental stages represent a critical window during which cells are highly sensitive to metabolic stress, enabling long-lasting adaptations that influence cellular function and stress resilience later in life. During development, environmental perturbations such as nutrient imbalance act as chronic metabolic stressors that reprogram cellular metabolism, survival pathways, and stress-response signalling, with consequences that persist beyond the initial insult 1.

This concept aligns with developmental programming models, including Barker’s “fetal origins of adult disease” hypothesis, which propose that early metabolic stress induces stable cellular and molecular reprogramming that alters vulnerability to later challenges 2. Such adaptive responses to nutrient scarcity, often described as “thrifty” or “parsimonious” phenotypes, enhance short-term survival but may impose trade-offs by constraining metabolic flexibility and stress tolerance under subsequent insults. Thus, metabolic balance during development is critical for preserving cellular adaptability and maintaining appropriate stress-response capacity. Cardiac development and function critically depend on tightly regulated energy production and stress-responsive metabolic signalling. Cardiomyocytes utilize multiple energy substrates, and shifts in substrate preference during development represent adaptive responses to changing metabolic and oxygen availability 3. During early cardiac development, glucose represents a major energy substrate, yet its availability is tightly regulated and often limited, creating a metabolically constrained environment. Glucose uptake is mediated by glucose transporters (GLUTs), which play a central role in determining cellular energy availability and stress sensitivity. Early cardiac development also occurs under relative hypoxia, stabilizing hypoxia-inducible factor-1 α (HIF-1 α ), a key regulator of metabolic adaptation and cellular stress responses 4. Disturbances in fetal glucose metabolism, such as diabetic pregnancy, are associated with congenital heart disease 5. Clinically, maternal dysglycemia is highly prevalent—gestational diabetes affects ~10–15% of pregnancies and neonatal hypoglycemia remains one of the most common metabolic complications at birth—underscoring the need to understand how early glucose environments shape cardiac development and long-term disease risk 6, 7. Around birth, cardiomyocytes transition from glucose utilization to fatty acid oxidation, adapting to postnatal energetic demands 8. This switch requires upregulation of key proteins, including the fatty acid transporter CD36 and carnitine palmitoyltransferase 1 (CPT1), and its dysregulation has been linked to adult cardiometabolic disease.

However, despite clear evidence linking maternal glycemic status to offspring cardiac outcomes, it remains unclear how specific glucose environments during cardiomyocyte differentiation directly program metabolic transporters and stress-response pathways. H9c2 embryonic rat cardiomyoblasts provide a tractable in vitro system to address this gap. Importantly, in the context of this study, H9c2 cells are not intended to model mature cardiomyocyte physiology, but rather to recapitulate early-stage cardiomyoblast differentiation, making them particularly suitable for investigating developmental metabolic programming under defined glycemic conditions. Although they do not fully recapitulate adult cardiomyocytes, H9c2 cells undergo partial cardiac differentiation and exhibit fetal-like metabolic remodelling, including upregulation of mitochondrial and β -oxidation pathways 9, 10. These cells are widely used to investigate cardiomyocyte development and stress responses, and their energetic profiles are closer to primary cardiomyocytes than many alternative models 11.

Here, we used differentiating H9c2 cardiomyoblasts to model exposure during pregnancy to hypoglycemia, normoglycemia, and hyperglycemia, and examined their effects on cardiac metabolic markers and survival pathways. Differentiated cells were also subjected to anoxia–reoxygenation to test cell stress resilience. We hypothesized that glycemic stress during differentiation programs metabolic transport, mitochondrial stress responses, and the autophagy–apoptosis axis, thereby altering tolerance to anoxia–reoxygenation stress and providing mechanistic insight into the developmental origins of cardiac vulnerability. To our knowledge, this is the first study to directly compare hypoglycemic and hyperglycemic programming on cardiomyocyte stress adaptation during differentiation.

## RESULTS

To simulate a condition of maternal malnutrition during fetal development, cardiomyoblast cells of the H9c2 line were subjected to different glucose concentrations during differentiation into cardiomyocytes. After differentiation, we evaluated cellular morphology, metabolic transporter expression, lipid accumulation, and stress-response pathways.

### Characterization and differentiation of the H9c2 cell line

To validate cardiomyocyte cell differentiation under physiological conditions, phase-contrast microscope images were first acquired to observe morphological differences between the two cell groups. The results shown in Figure 1**A** did not show significant differences in the roundness index, but differentiated cells (II) displayed a greater tendency to form cylinders and elongated shapes than undifferentiated cells (I).

mRNA and protein expression of cardiac troponin T (TnTc) (Figure 1**B**–**D**) were then assessed. TnTc mRNA levels were significantly increased in differentiated versus undifferentiated cells. We found that TnTc is not only more highly expressed in differentiated than undifferentiated cells, but as Figure 1**D** shows, there is an organizational change of the protein in differentiated H9c2 cardiomyoblasts. Accordingly, differentiated cells display a distinct striated-like distribution of TnTc, with well-aligned filamentous structures resembling a sarcomeric organization, whereas undifferentiated cells exhibit only diffuse cytoplasmic staining, consistent with the acquisition of a cardiomyocyte-like phenotype.

**Figure 1:**
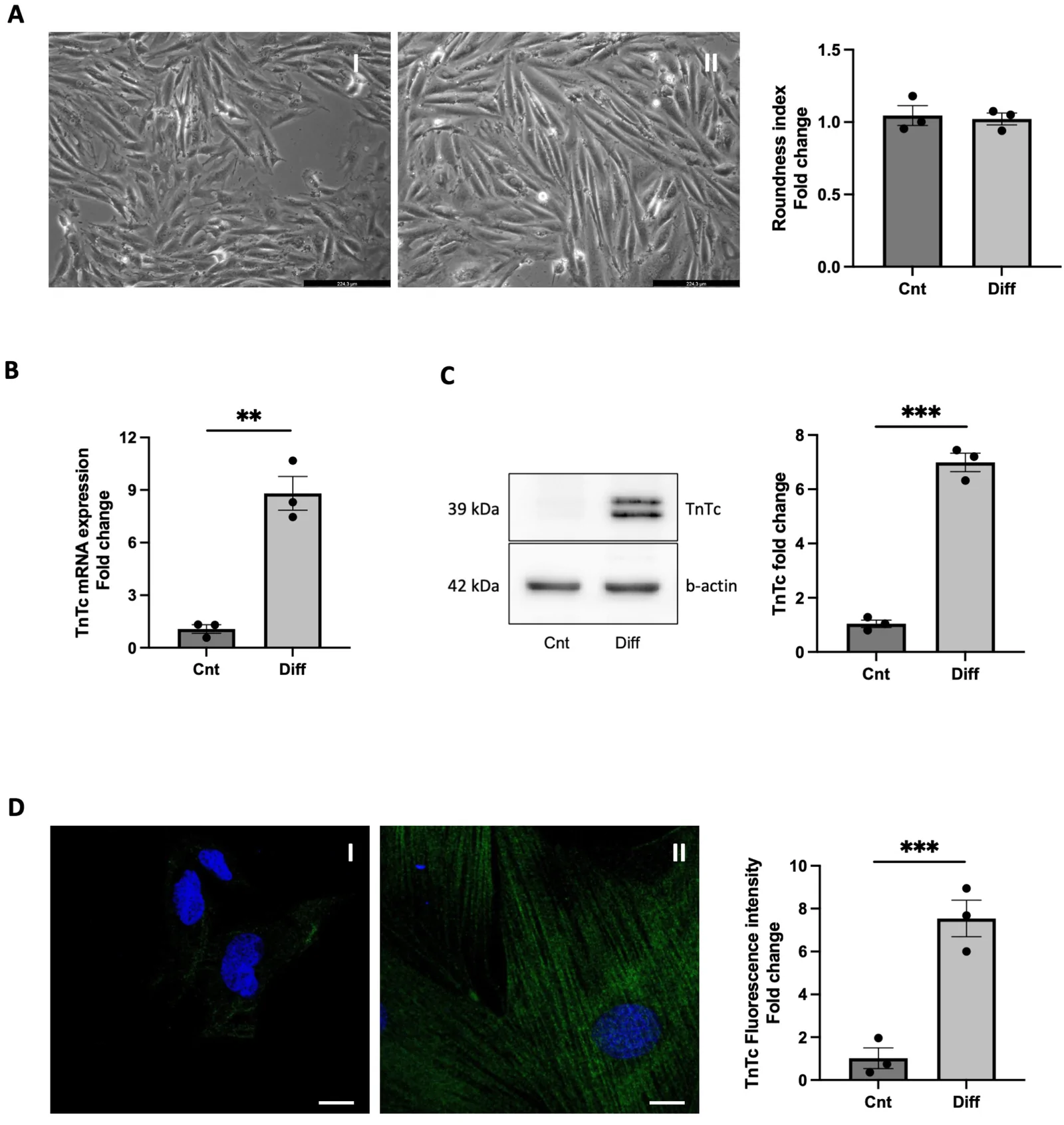
Differentiation of the H9c2 cell line. **(A)** Representative phase contrast images of undifferentiated control cells and differentiated cells into I and II, respectively; data represent three independent biological experiments (n=3), 9 images per condition, original magnification 10X, bar=224,3 μ m. **(B)** TnTc mRNA expression in differentiated H9c2 (Diff) compared to undifferentiated (Cnt); data represent three independent biological experiments (n=3), each performed with technical replicates, (^⁎⁎^p < 0.01). **(C)** TnTc protein expression in differentiated H9c2 (Diff) compared to undifferentiated (Cnt); TnTc was normalized to β -actin; data represent three independent biological experiments (n=3) (^⁎⁎⁎^p < 0.001). **(D)** Representative images and relative fluorescence intensity graph of TnTc in undifferentiated (I) H9c2 and differentiated (II) H9c2. TnTc marked in green, with blue DAPI, the nucleus; data represent three independent biological experiments (n=3), 9 images per condition, original magnification 63X, bar=15 μ m; (^⁎⁎⁎^p < 0.001). Statistical analysis by unpaired two-tailed Student’s t-test. All data are reported as means ± SEM.

### Effects of glucose on cardiac differentiation

To evaluate whether glucose availability affects H9c2 differentiation, cells were cultured for six days under hypoglycemic, normoglycemic, or hyperglycemic conditions. Morphological analysis revealed slight differences in cell shape among the experimental groups. Quantitative assessment using the roundness index showed a significant reduction exclusively in cells differentiated under hyperglycemic conditions compared with normoglycemic controls, whereas no significant differences were observed between hypoglycemic and normoglycemic conditions (Figure 2**A**). Cell viability, measured at the end of the differentiation period by SRB assay, did not differ significantly among groups, suggesting that glucose concentration did not affect cell survival during differentiation (Figure 2**B**). The gene expression of the cardiac marker TnTc was also examined. A significant upregulation of TnTc mRNA levels was observed in hyperglycemic conditions compared to normoglycemia, while the hypoglycemic group showed no significant difference versus normoglycemia (Figure 2**C**). Overall, glucose availability influenced differentiation marker expression without affecting cell viability.

**Figure 2:**
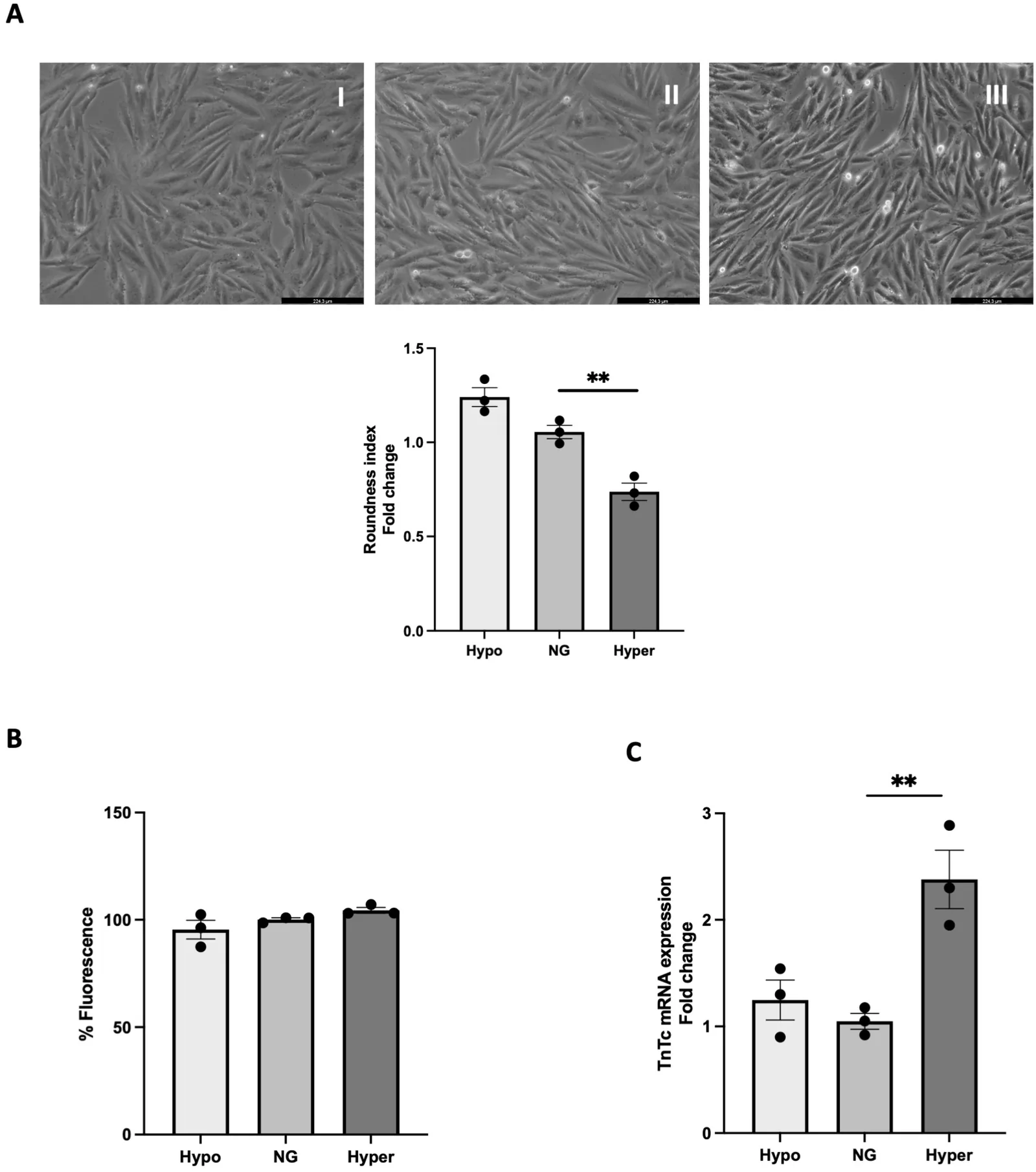
Differentiation of the H9c2 cell line under different glucose concentrations. **(A)** Representative phase-contrast images of H9c2 cells differentiated under hypoglycemic (Hypo; I), normoglycemic (NG; II), or hyperglycemic (Hyper; III) conditions. Quantification of the roundness index revealed a significant decrease in Hyper-treated cells compared with NG, indicating a more elongated morphology consistent with cardiomyocyte differentiation (^⁎⁎^p < 0.01). Scale bars = 200 μ m. **(B)** Cell viability was assessed by the SRB assay after differentiation under the three glucose conditions. No significant differences in cell viability were observed among Hypo, NG, and Hyper groups, indicating that the glucose concentrations used did not affect cell survival. Data represent three independent biological experiments (n=3), each performed with 9 technical replicates. **(C)** Relative mRNA expression of cardiac troponin T (TnTc) measured by quantitative real-time PCR. Hyperglycemic conditions significantly increased TnTc expression compared with NG, suggesting enhanced expression of a cardiac differentiation marker under high-glucose conditions (^⁎⁎^p < 0.01). Gene expression was normalized to the housekeeping gene and expressed as fold change relative to NG. All data are presented as mean ± SEM of three independent experiments. Statistical significance was determined by one-way ANOVA followed by Dunnett’s multiple comparisons test.

### Effects of glucose on cardiac metabolism

We next examined metabolic transporter expression under each glucose condition. Protein levels of the glucose transporter GLUT1 and the glucose uptake ability of the differentiated H9c2 cells were evaluated.

Densitometric analysis showed significant differences in GLUT1 expression among treatments (Figure 3**A**). Compared to normoglycemic treatment, GLUT1 levels decreased in hyperglycemia and increased under hypoglycemia. To determine whether changes in GLUT1 protein expression were associated with functional alterations in glucose utilization, glucose uptake was evaluated over a 2-h incubation period. As shown in Figure 3**B**, hypoglycemic cells displayed a significantly higher glucose uptake than normoglycemic controls, whereas hyperglycemic cells exhibited a significantly lower glucose uptake. The present findings are consistent with the modulation of GLUT1 protein expression observed under varying glucose conditions (Figure 3**A**). This observation indicates that glucose availability modulates not only GLUT1 expression but also the capacity of differentiated H9c2 cells to take up glucose. This, in turn, suggests adaptations to the extracellular glucose environment.

Furthermore, the fatty-acid transporter CD36 protein and the expression levels of the fatty-acid oxidation enzyme CPT1 were assessed, in conjunction with the degree of lipid accumulation. Protein expression levels of the CD36 fatty acid transporter were significantly increased in conditions of hypoglycemia and hyperglycemia compared with normoglycemia, as shown in the representative graph (Figure 4**A**). These findings were further supported by increased CD36 mRNA expression in both conditions and immunofluorescence staining, which also revealed enhanced membrane localization of CD36 under both hypoglycemic and hyperglycemic conditions (Supplementary Figure S1).

**Figure 3:**
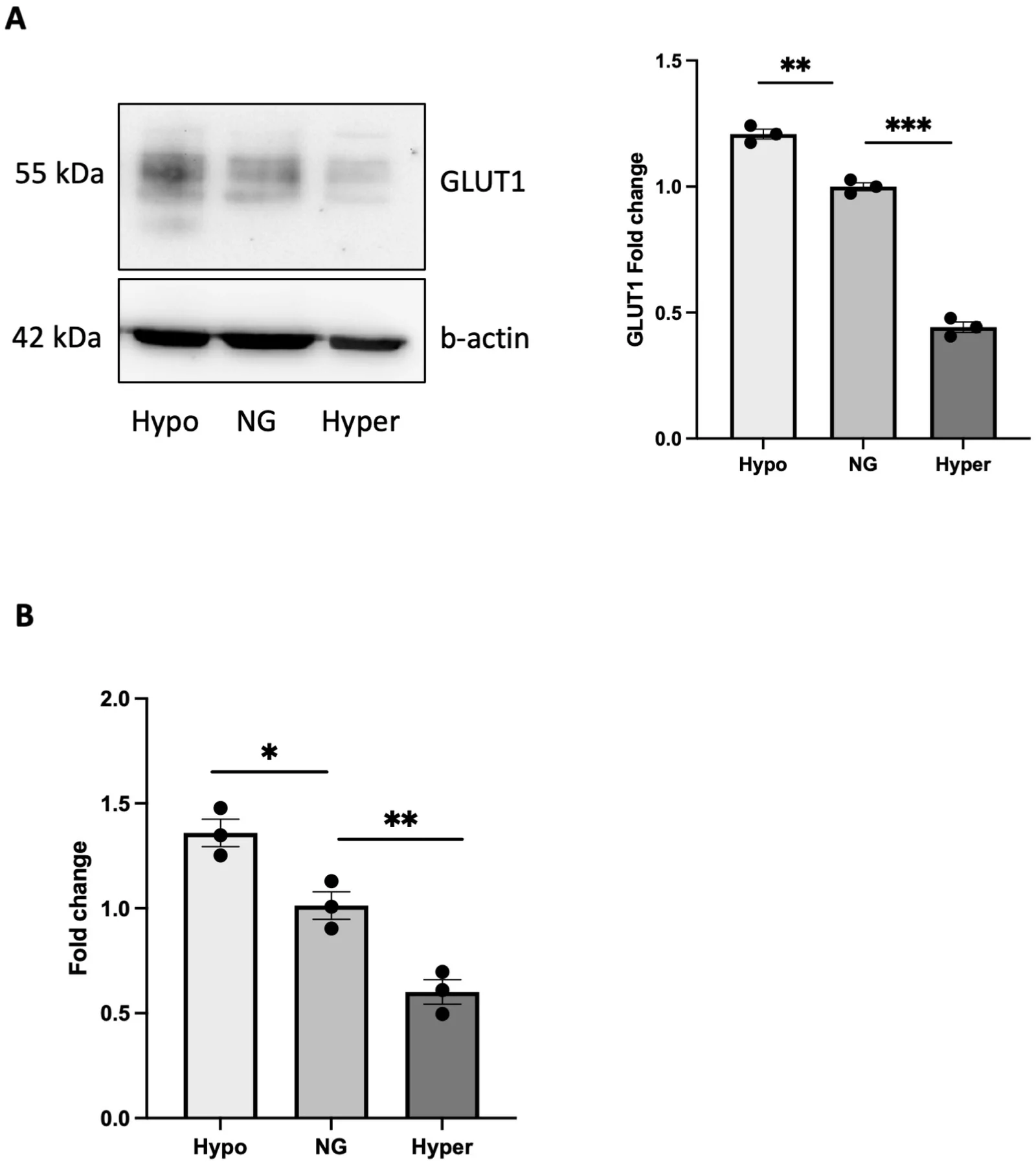
Effects of glucose availability on GLUT1 expression and glucose uptake in differentiated H9c2 cardiomyoblasts. **(A)** Representative Western blot and densitometric analysis of GLUT1 protein expression in H9c2 cells differentiated under hypoglycemic (Hypo), normoglycemic (NG), or hyperglycemic (Hyper) conditions. GLUT1 protein levels were significantly increased in Hypo cells compared with NG (^⁎⁎^p < 0.01) and were significantly reduced in Hyper cells compared with NG (^⁎⁎⁎^p < 0.001). β -actin was used as the loading control. **(B)** Glucose uptake assay performed over a 2-h incubation period following differentiation under Hypo, NG, or Hyper conditions. Before the assay, cells from all experimental groups were incubated for 2 h in the same glucose-containing medium. Glucose uptake was determined by measuring the decrease in glucose concentration in the culture medium and expressed as a fold change relative to NG. Hypo-treated cells exhibited significantly greater glucose uptake than NG (^⁎^p < 0.05), whereas Hyper-treated cells showed significantly reduced glucose uptake (^⁎⁎^p < 0.01). Data represent three independent biological experiments, each performed with 9 technical replicates. All data are presented as mean ± SEM of three independent experiments. Statistical significance was determined by one-way ANOVA followed by Dunnett’s multiple comparisons test.

Regarding the protein expression levels of CPT1, we observed an increase in hypoglycemia compared to differentiated cells in normoglycemia. In contrast, no change was observed in hyperglycemia, as shown in Figure 4**B**, indicating an enhanced fatty acid transport under hyperglycemic conditions.

**Figure 4:**
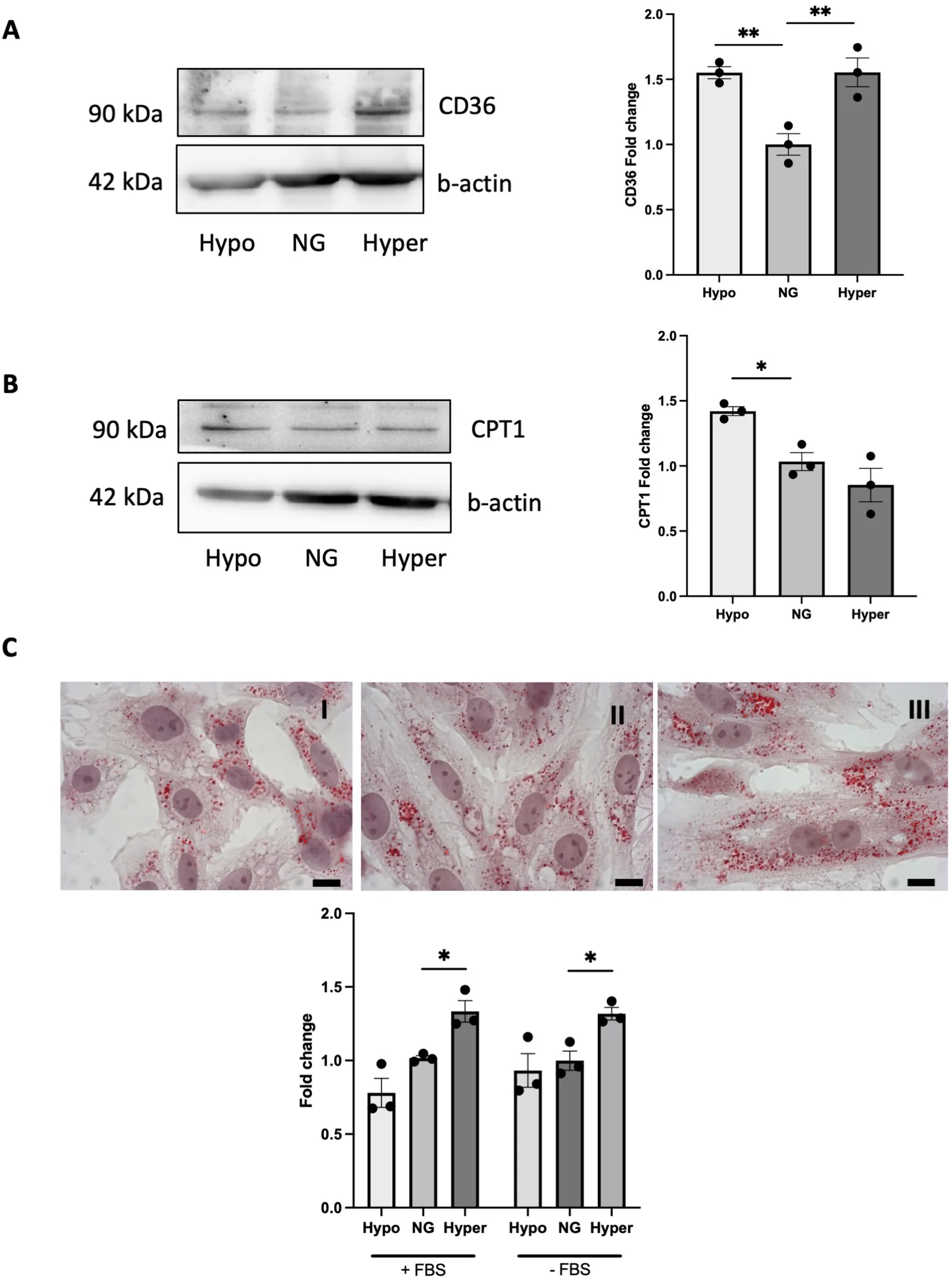
CD36 and CPT1 expression and Lipid accumulation in differentiated H9c2. **(A)** Representative Western blot and densitometric analysis of CD36 protein expression in H9c2 cells differentiated under hypoglycemic (Hypo), normoglycemic (NG), or hyperglycemic (Hyper) conditions. CD36 expression was significantly increased in both Hypo- and Hyper-treated cells compared with NG. β -actin was used as a loading control (^⁎⁎^p < 0.01). **(B)** Representative Western blot and densitometric analysis of carnitine palmitoyltransferase-1 (CPT1). CPT1 protein levels were significantly increased in Hypo compared with NG. In contrast, Hyper did not differ significantly from NG. β -actin served as a loading control (^⁎^p < 0.05). **(C)** Representative Oil Red O staining showing intracellular neutral lipid accumulation in differentiated H9c2 cells cultured under Hypo (I), NG (II), and Hyper (III) conditions. Quantification of Oil Red O staining performed in the presence (+FBS) or absence ( − FBS) of fetal bovine serum (FBS) demonstrated a significant increase in lipid accumulation in Hyper-treated cells under both experimental conditions. Data are expressed as fold change relative to each NG. Values represent mean ± SEM of three independent experiments (N = 3). Statistical significance was determined by one-way ANOVA followed by Dunnett’s multiple comparisons test.

Because CD36 and CPT1 expression changed with glucose levels, we assessed intracellular lipid accumulation. Oil Red O staining and spectrophotometric quantification were performed to measure intracellular lipid accumulation in H9c2 cells differentiated under different glucose conditions. Staining with Oil Red O, which acts selectively on neutral lipids, revealed a statistically significant increase in cytoplasmic accumulation of intracellular lipids in the hyperglycemic treatment, as shown in the optical microscopy images (Figure 4**C-III**) and the absorbance graph. In contrast, compared to the normoglycemic group, no changes in lipid accumulation were observed under hypoglycemic conditions (Figure 4**C-I**). Importantly, a similar pattern was observed when the post-differentiation FBS challenge was removed (images not shown), confirming that the increased lipid accumulation in hyperglycemia-conditioned cells was primarily associated with prior glycemic exposure rather than serum lipid loading (Figure 4**C**, right graph).

### Effects of anoxia-reoxygenation on cell viability

To assess the impact of A/R on cell survival under different glycemic conditions, cell viability was evaluated by both SRB and MTT assays. Both assays consistently showed a significant reduction in cell viability in hypoglycemic and hyperglycemic conditions compared with normoglycemic cells following A/R (Supplementary Fig. S3). These findings indicate that altered glucose availability exacerbates cellular vulnerability to A/R injury, with both glucose deprivation and excess contributing to impaired cell survival.

To investigate the mechanisms underlying this susceptibility to A/R, we next analyzed autophagic and apoptotic responses. Basal autophagic and apoptotic marker levels were comparable among the experimental groups before A/R (Supplementary Fig. S2), indicating that glycemic conditioning did not alter the basal status of these pathways.

Following A/R, autophagy was differentially regulated according to the glucose concentration used during differentiation. Cells differentiated under hypoglycemic conditions exhibited a significant increase in the LC3B-II/LC3B-I ratio compared with normoglycemic cells (Figure 5**A**), indicating enhanced autophagosome formation. In contrast, no significant increase in LC3B-II accumulation was observed in hyperglycemic cells. Concerning the p62 expression levels, these remained constant under all conditions investigated in this study (Figure 5**A**).

To determine whether the increased LC3B-II accumulation observed under hypoglycemic conditions reflected enhanced autophagic flux rather than impaired lysosomal degradation, differentiated H9c2 cells were treated with chloroquine (CQ), an inhibitor of lysosomal acidification. In the absence of CQ, hypoglycemic cells displayed a significantly higher LC3B-II/LC3B-I ratio compared with normoglycemic cells, while p62 levels remained comparable among all experimental groups (Figure 5**A**), suggesting increased autophagosome formation without evidence of impaired autophagic degradation. Following CQ treatment, LC3B-II levels statistically increased in hypoglycemic and normoglycemic conditions, and only a trend is appreciable in hyperglycemic conditions (Figure 5**B**, right graph), consistent with the expected accumulation resulting from lysosomal inhibition. However, only hypoglycemic cells exhibited a marked and significant accumulation of p62 compared with untreated cells (Figure 5**B**, left graph). Since p62 is selectively degraded through autophagy, its accumulation upon CQ-mediated lysosomal blockade indicates an increased dependence on autophagic degradation under hypoglycemic conditions. Conversely, the lack of a comparable p62 increase in normoglycemic and hyperglycemic cells suggests that autophagic flux is not similarly enhanced in these conditions. Together, these findings support the conclusion that hypoglycemia stimulates a functional autophagic response characterized by increased autophagosome turnover, whereas hyperglycemia does not induce a comparable increase in autophagic flux after A/R treatment.

**Figure 5:**
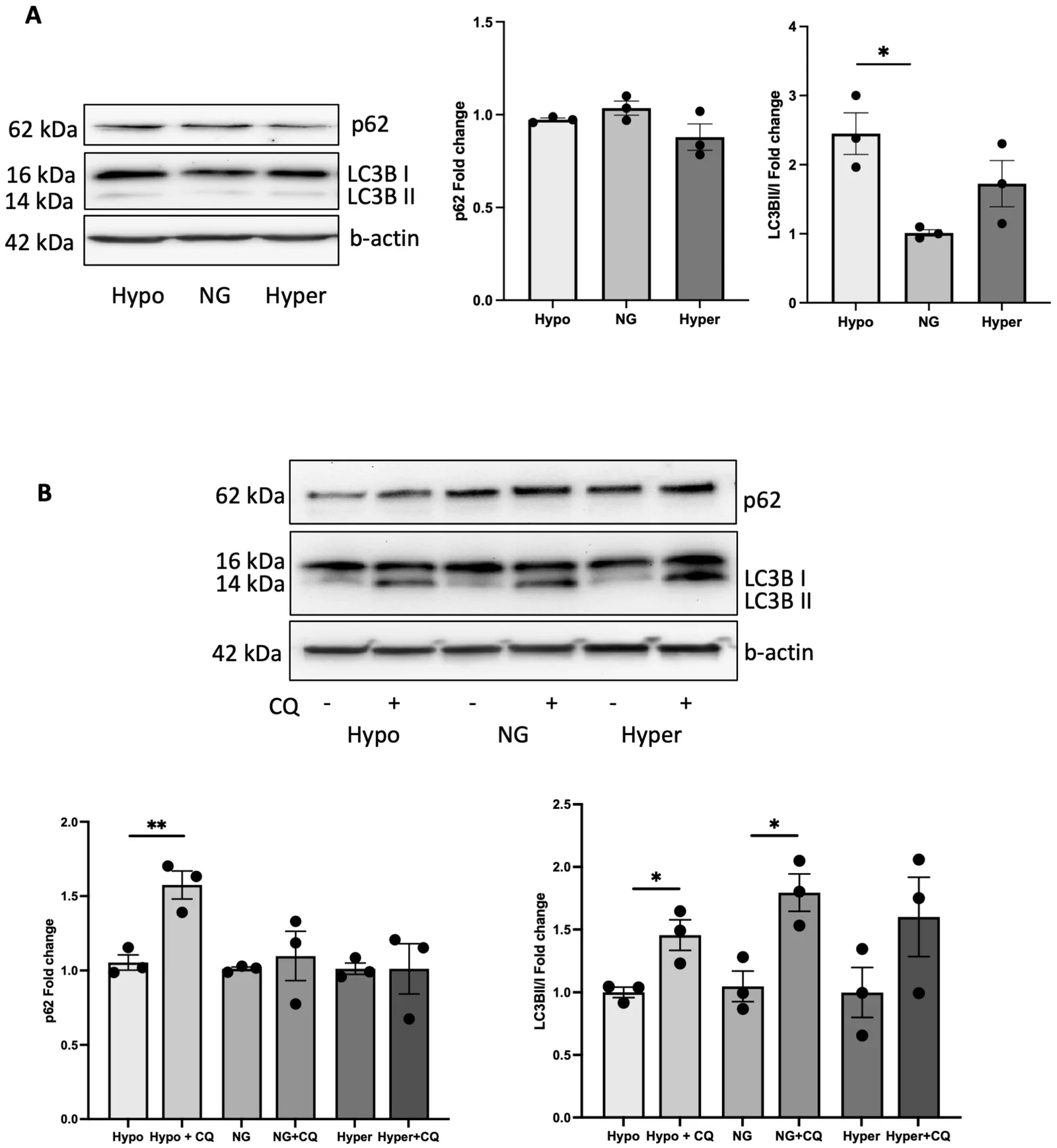
Assessment of autophagic flux in differentiated H9c2 cells under different glucose conditions. **(A)** Representative Western blots and densitometric analyses of the LC3B-II/LC3B-I ratio (^⁎^p < 0.05) and p62 protein expression in differentiated H9c2 cells cultured under hypoglycemic, normoglycemic, or hyperglycemic conditions after A/R. Statistical significance was determined by one-way ANOVA followed by Dunnett’s multiple comparisons test. **(B)** Representative Western blots and densitometric analyses of the LC3B-II/LC3B-I ratio and p62 protein expression (^⁎^p < 0.05, ^⁎⁎^p < 0.01) in differentiated H9c2 cells cultured under hypoglycemic, normoglycemic, or hyperglycemic conditions in the absence ( − CQ) or presence (+CQ) of chloroquine (CQ, 25 μ M). Statistical analysis was performed using an unpaired two-tailed Student’s t-test. β -actin served as a loading control. Data are presented as mean ± SEM of three independent experiments ( N=3 ).

To further investigate whether altered glucose availability affected cell fate following A/R, we evaluated the activation of apoptotic pathways by assessing caspase-3 and PARP cleavage. Hyperglycemic cells showed a significant increase in cleaved caspase-3 levels compared with normoglycemic conditions, accompanied by an increased ratio of cleaved to full-length PARP (Figure 6**A**), indicating enhanced activation of the apoptotic machinery.

To confirm these findings by an independent approach, apoptotic cell death was assessed by TUNEL staining. Consistent with the biochemical analysis, hyperglycemic cells displayed a marked increase in the percentage of TUNEL-positive nuclei (Figure 6**B**, left panel). Interestingly, hypoglycemic cells also exhibited a moderate increase in TUNEL-positive nuclei, although this effect was considerably lower than that observed under hyperglycemia. Indeed, quantitative analysis revealed approximately 1% TUNEL-positive nuclei in normoglycemic cells, increasing to ~6% under hypoglycemic conditions and reaching ~14% in hyperglycemic cells (Figure 6**B**, right graph). Together, these findings demonstrate that hyperglycemic differentiation conditions enhance the susceptibility of H9c2 cells to A/R-induced apoptotic damage, as evidenced by both activation of apoptotic signaling markers and increased DNA fragmentation.

**Figure 6:**
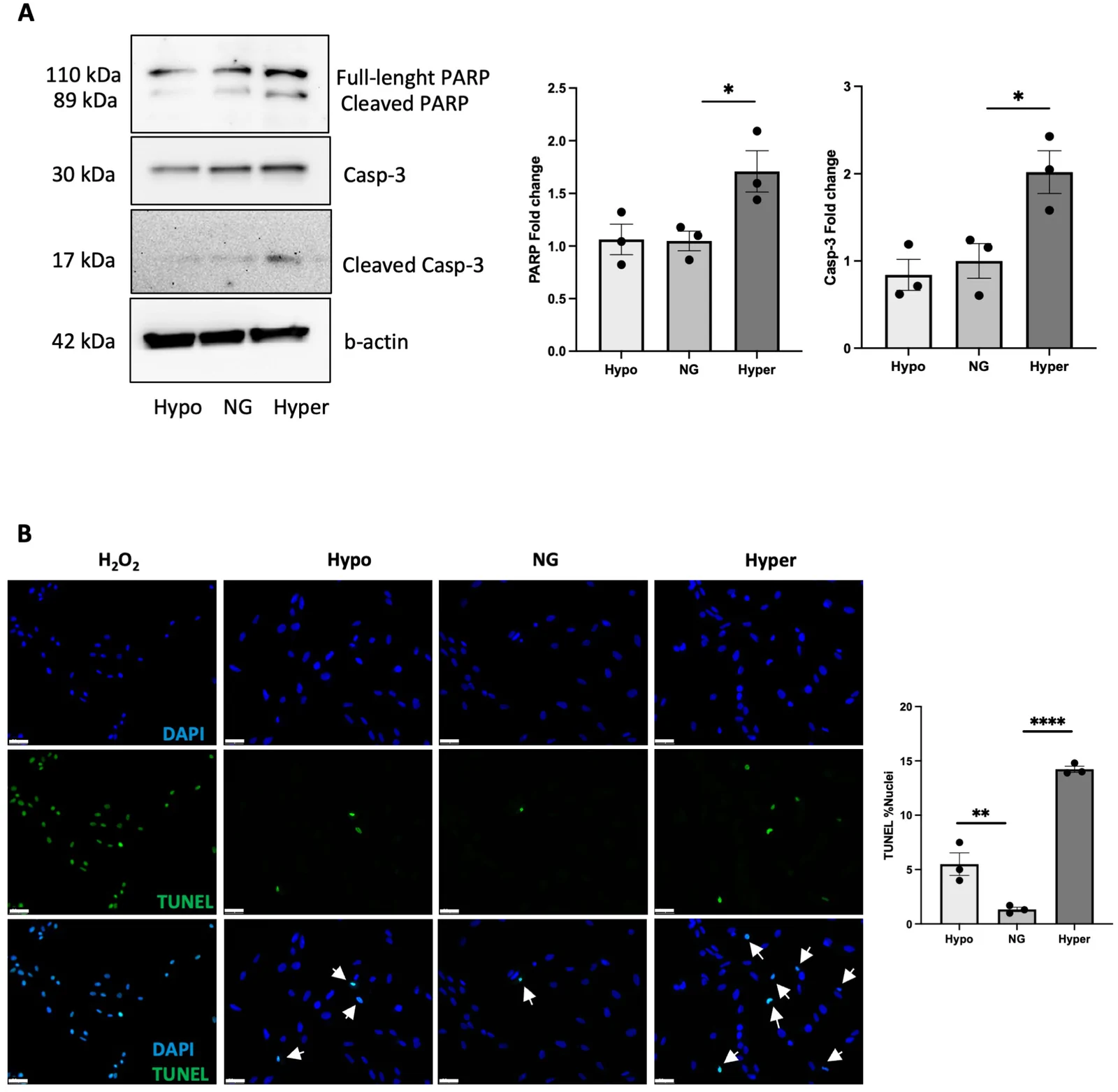
Hyperglycemic conditions promote apoptosis in differentiated H9c2 cells following anoxia/reoxygenation. **(A)** Representative Western blots and densitometric analyses of full-length and cleaved PARP ratio, as well as full-length and cleaved caspase-3 ratio, in differentiated H9c2 cells cultured under hypoglycemic (Hypo), normoglycemic (NG), or hyperglycemic (Hyper) conditions following anoxia/reoxygenation. Hyperglycemic cells exhibited increased levels of cleaved PARP and cleaved caspase-3 compared with normoglycemic conditions (^⁎^p < 0.05). β -actin was used as a loading control. **(B)** Representative fluorescence images showing TUNEL staining (green) and nuclear counterstaining with DAPI (blue) in differentiated H9c2 cells under the indicated glucose conditions after A/R treatment. Hydrogen peroxide-treated cells (H ${}_{2}$ O ${}_{2}$ ) were used as a positive control for apoptosis induction. White arrows indicate TUNEL-positive nuclei. Scale bar = 50 μ m. Quantification of apoptotic cells is expressed as the percentage of TUNEL-positive nuclei relative to total DAPI-positive nuclei (^⁎⁎^p < 0.01; ^⁎⁎⁎⁎^p < 0.0001). Data are presented as mean ± SEM of three independent experiments ( N=3 ). Statistical analysis was performed using one-way ANOVA followed by Dunnett’s multiple comparisons test.

## DISCUSSION

In this study, differentiating H9c2 cardiomyoblasts were used to examine how glycemic environments influence metabolic programming and stress resilience. Both hypoglycemia and hyperglycemia altered the expression of key transporters, including GLUT1, CD36, and CPT1, reshaping substrate utilization pathways. Hypoglycemia upregulated GLUT1, CD36, and CPT1, indicating enhanced glucose uptake, whereas hyperglycemia suppressed GLUT1 and selectively increased CD36 without promoting CPT1, suggesting enhanced lipid uptake but inefficient oxidation. Consistent with this imbalance, hyperglycemia-differentiated cells exhibited a marked intracellular lipid accumulation, indicating that excessive fatty acid influx may exceed mitochondrial oxidative capacity when CPT1 expression is not concomitantly upregulated. This phenotype supports the possibility of lipid overload and ensuing lipotoxic stress. Under anoxia–reoxygenation, hypoglycemia-differentiated cells showed enhanced autophagic responses, whereas hyperglycemia-differentiated cells displayed increased apoptotic signaling, revealing distinct stress-adaptation profiles. These findings align with evidence that intrauterine nutrient imbalances disrupt cardiac development and metabolism, contributing to lifelong cardiovascular risk 2, 12. To our knowledge, this is the first study to directly compare hypoglycemia and hyperglycemia during H9c2 cardiomyoblast differentiation, uncovering glycemia-dependent metabolic adaptations and stress-response pathways that parallel the fetal-to-postnatal metabolic transition.

Furthermore, our findings hold translational relevance, as neonatal hypoglycemia and maternal hyperglycemia are common clinical conditions associated with long-term cardiovascular risk. By showing that altered glucose availability during differentiating H9c2 cardiomyoblasts programs distinct metabolic and stress-response pathways, our model provides mechanistic insight into how early glycemic instability may predispose the developing heart to later dysfunction. Together, these results highlight a critical developmental window during which glucose levels can influence postnatal cardiac resilience.

Despite increasing clinical evidence, the cellular mechanisms by which early glycemic disturbances shape cardiomyocyte maturation and stress vulnerability remain insufficiently defined. Since metabolic substrate availability determines energy homeostasis, glycemic conditions during differentiation may critically influence cardiomyocyte survival pathways. Excess glucose promotes oxidative stress and apoptosis in cardiac cells 13, while glucose deprivation mimics ischemia, inducing autophagy and, if unresolved, apoptosis 14–16. These metabolic stresses are primarily coordinated by pathways such as AMPK–mTOR, HIF-1 α , and ROS-mediated mitochondrial signaling, which regulate the balance between adaptive autophagy and maladaptive apoptosis. Importantly, metabolic flexibility strongly influences hypoxia tolerance by determining mitochondrial efficiency and oxidative stress burden, yet it remains unclear how developmental glycemic stress shapes this. Maternal nutrition is a key regulator of embryonic and neonatal cardiac development, partly mediated by epigenetic reprogramming consistent with Barker’s “fetal origins” hypothesis 17. In the fetal heart, glucose is the primary substrate, and glycolysis predominates in ATP production 18, 19. Disruption of glucose availability during gestation, either excess or deficiency, perturbs metabolic programming and increases susceptibility to cardiovascular disease in later life. Maternal hyperglycemia is linked to heart failure, obesity, and metabolic dysfunction in offspring 20, 21, whereas maternal hypoglycemia predisposes to congenital anomalies, including cardiac malformations 22. Tight maternal glycemic control mitigates these risks 23.

GLUT1 is highly expressed during fetal development and declines postnatally 24, 25. In our model, hypoglycemia enhanced GLUT1, whereas hyperglycemia suppressed it, suggesting feedback-sensitive regulation. Consistently, functional assessment of glucose uptake revealed increased glucose utilization under hypoglycemic conditions and reduced glucose handling following hyperglycemic conditioning, supporting the presence of glucose-dependent metabolic adaptations induced by chronic glycemic stress. The developmental shift toward fatty acid oxidation was mirrored by CD36 and CPT1 upregulation; hypoglycemia amplified both proteins, while hyperglycemia selectively increased CD36, indicating potential lipid accumulation and lipotoxic risk 26. Indeed, maternal metabolic conditions such as gestational diabetes are associated with fetal dyslipidemia 27. Consistent with these observations, hyperglycemia-differentiated H9c2 cells showed increased intracellular lipid accumulation, suggesting that inadequate coupling between fatty acid uptake and mitochondrial oxidation may contribute to metabolic stress. Importantly, additional iso-osmotic control experiments performed in the presence of mannitol yielded results comparable to those obtained under standard experimental conditions, indicating that the observed alterations are primarily associated with differences in glucose availability rather than nonspecific osmotic stress.

Hypoxia–reoxygenation reduced viability under both conditions, reflecting increased vulnerability to oxidative and metabolic stress 28, 29. In the present study, the Anoxia/Reoxygenation (A/R) protocol was selected to evaluate differential adaptive responses induced by prior glycemic conditioning, including the activation of autophagic and apoptotic pathways. Mechanistically, hypoglycemia appeared to preferentially promote autophagic responses, as indicated by an increased LC3B-II/LC3B-I ratio following A/R exposure. Importantly, chloroquine treatment further enhanced LC3B-II accumulation together with p62 levels under hypoglycemic conditions, supporting the presence of an active autophagic flux rather than impaired autophagosome clearance. These findings suggest that hypoglycemia-conditioned cells engage adaptive autophagic mechanisms in response to metabolic and hypoxic stress. In contrast, hyperglycemia promoted apoptotic cell death following A/R, as demonstrated by the coordinated increase in caspase-3 activation, PARP cleavage, and TUNEL-positive nuclei. Consistent with these findings, both MTT and SRB assays revealed a significant reduction in cell viability, indicating that hyperglycemic conditioning markedly increases cellular vulnerability to A/R injury. This phenotype likely reflects impaired mitochondrial integrity and a lowered apoptotic threshold. Furthermore, lipid accumulation under hyperglycemic conditions may aggravate mitochondrial dysfunction, thereby amplifying apoptotic signaling. These findings highlight how opposite metabolic challenges—nutrient deprivation versus nutrient excess—differentially modulate the equilibrium between adaptive and maladaptive stress-response pathways in differentiating H9c2 cardiomyoblasts.

Limitations of this study include the use of H9c2 cells, which do not fully replicate the structure or metabolism of adult cardiomyocytes. However, in the context of this study, they provide a relevant model of differentiating cardiomyoblasts with fetal-like metabolic features, suitable for investigating early-stage metabolic programming under controlled glycemic conditions. Here, we modelled constant glucose exposure, although in vivo the maternal-fetal glucose environment is dynamic and modulated by hormonal and placental signals. Moreover, we focused on transporter expression and cell death pathways, without directly assessing upstream signaling or mitochondrial bioenergetics. Future studies should integrate metabolic flux analysis, mitochondrial respiration assays, and *in vivo* models to dissect the developmental programming of cardiac metabolism further.

In summary, both hypoglycemia and hyperglycemia during H9c2 cardiomyoblast differentiation impair resilience to hypoxic stress by differentially modulating autophagic and apoptotic pathways, with hypoglycemic conditions favoring adaptive autophagic responses and hyperglycemic conditions associated with enhanced apoptotic signaling. These findings provide novel evidence that early-life nutrient imbalances differentially program cardiomyocyte fate, emphasizing the critical role of metabolic homeostasis in shaping adaptive versus maladaptive responses. Understanding these pathways may support strategies to optimize maternal glycemic control and improve neonatal metabolic outcomes, highlighting metabolic flexibility as a target for reducing lifelong cardiovascular vulnerability.

## MATERIALS AND METHODS

### Cell culture and differentiation

The H9c2 cell line was used in this study, which is derived from embryonic cardiomyoblasts of rat embryonic heart (ATCC® CRL 1446™, Manassas, VA, USA). Cells were cultured in 75 cm ${}^{2}$ flasks with DMEM medium (Dulbecco’s Modified Eagle Medium) with 4.5 g/L glucose, enriched with 10% (v/v) fetal bovine serum (FBS) (Biochrom, Berlin, Germany), 1% (v/v) L-glutamine (2 mM; Sigma Chemical Co) and 1% (v/v) Penicillin/Streptomycin. The flasks were incubated at 37 ${}^{\circ }$ C with 97% humidity and 5% CO ${}_{2}$ until 70% confluence was reached.

H9c2 cells were differentiated *in vitro* using the culture medium with 1% FBS and 1 μ M retinoic acid (R2625, Sigma-Aldrich, St. Louis, MO, USA) 9, 30. During the 6-day differentiation period, cells were simultaneously exposed to different glucose concentrations: 2.5 mM (hypoglycemia), 5 mM (normoglycemia), or 30 mM (hyperglycemia). To exclude the contribution of osmotic stress, D-mannitol (M4125, Sigma-Aldrich) was added to the hypoglycemic and normoglycemic media so that the combined concentration of glucose and mannitol was 30 mM in all experimental groups. Accordingly, 27.5 mM D-mannitol was added to the 2.5 mM glucose medium, and 25 mM D-mannitol was added to the 5 mM glucose medium, whereas no mannitol was added to the 30 mM glucose condition. The culture medium was renewed daily, and cells were analyzed after 6 days of differentiation. Glucose concentrations were selected based on physiological and pathological ranges commonly used to model glycemic stress in vitro. A concentration of 5 mM was used to represent normoglycemia, reflecting physiological glucose levels in rodents and standard conditions for cardiac cell culture. A reduced concentration of 2.5 mM was used to model hypoglycemia and nutrient restriction, whereas 30 mM glucose was used to model the metabolic stress associated with diabetic and gestational hyperglycemia 13, 31–33.

### Sulforhodamine B (SRB) assay

H9c2 cells were seeded on a 96-well plate at a density of 2 × 10 ${}^{3}$ cells/well for differentiation. At the end of the treatments, the culture medium was removed, and cells were gently washed three times with phosphate-buffered saline (PBS). Cells were then fixed by adding 10% (w/v) cold trichloroacetic acid (TCA; 100 μ L/well) and incubated for 30 min at 4 ${}^{\circ }$ C. After fixation, wells were washed three times with distilled water, and plates were air-dried. Fixed cells were stained with 0.4% (w/v) SRB solution (100 μ L/well) for 30 min at room temperature, protected from light. Excess dye was removed by four washes with 1% (v/v) acetic acid, and plates were air-dried. Protein-bound SRB was solubilized with 10 mM Tris base solution (200 μ L/well) for 30 min at room temperature, protected from light. Fluorescence was measured from the top using a microplate reader at an excitation wavelength of 488 nm and an emission wavelength of 585 nm as previously described 34. Nine well replicates were performed for each study condition.

### MTT assay

Cells were seeded on a 96-well plate at a density of 2 × 103 cells/well for differentiation. At the end of the treatments, the culture medium was removed, and cells were gently washed three times with phosphate-buffered saline (PBS). Cells were incubated for 4 h with fresh medium in the presence of 1.2 mM MTT (3-(4,5-dimethylthiazol-2-yl)-2,5-diphenyltetrazolium bromide) (Sigma-Aldrich). The medium was removed, 100 μ L of DMSO was added to each well to dissolve the blue formazan crystals, and the plates were incubated at 37 ${}^{\circ }$ C for 10 min. The absorbance of the formazan dye was then measured at 570 nm with a microplate reader (VersaMax Tunable Microplate Reader, Molecular Devices, San Jose, CA, USA) and used to calculate cell viability. Data were expressed as a percentage of the basal control.

### Glucose uptake assay

H9c2 cells were seeded on a 96-well plate at a density of 2 × 10 ${}^{3}$ cells/well for differentiation. Briefly, at the end of differentiation, the culture medium was replaced with the normoglycemic condition medium for all experimental groups, and glucose levels remaining in the culture supernatant were measured after 2 hours. The glucose content was measured after incubation with the Amplex Red Glucose Assay Kit® (Thermo Fisher Scientific) for 30 min, covered from light. The reaction was revealed by measuring the absorbance through a microplate reader at 560 nm. Nine well replicates were performed for each study condition.

### Oil red O assay

H9c2 cells were plated on coverslips (6 × 10 ${}^{4}$ cells/well) or on 96-well plates (2 × 10 ${}^{3}$ cells/well), differentiated as reported above and at the end of differentiation, treated with 10% FBS for 24 hours to induce lipid droplet formation. The Oil Red O stock solution was prepared by reconstituting 0.5 g of Oil Red O powder (O0625; Sigma Aldrich) in 100 ml of 100% isopropanol. The working solution of Oil Red O was prepared by adding 3 parts of Oil Red O stock solution to 2 parts of distilled water, after which the solution was filtered with Whatman filter paper. For fixation, cells were washed once with PBS and fixed with 4% formaldehyde for 20 minutes at room temperature. After fixation, the cells were washed twice with PBS and incubated with the Oil Red O working solution for 30 minutes. The cells were then washed 3 times with water to remove excess stain and incubated with hematoxylin for 3 minutes to stain the nuclei. After staining, the cells were washed, and the slides were mounted with aqueous mountant for observation under a LEICA DMRB microscope with AxioCam MRc5. Nine non-overlapping fields per condition were acquired from three independent experiments using identical microscope settings. For the quantification of droplets, cells were plated in 96 wells, with treatments conducted in replicates of nine wells. The protocol was followed as described, with a modification in the final stage, in which isopropanol was added to extract Oil Red O. The supernatant was subjected to optical density (OD) determination at 540 nm using a spectrophotometer.

### Anoxia/Reoxygenation (A/R)

H9c2 cells were seeded in 6-well plates at a density of 6 × 10 ${}^{4}$ cells/well and differentiated as described above. To mimic ischemia–reperfusion injury, differentiated cells were subjected to an anoxia/reoxygenation (A/R) protocol based on a previously published method 35. Briefly, the plates were incubated in the same normoglycemic fresh medium across all conditions. Cells were placed in an airtight hypoxia chamber, which was flushed with 100% nitrogen (N ${}_{2}$ ) to generate an anoxic atmosphere, and incubated for 2 h at 37 ${}^{\circ }$ C. Subsequently, the medium was replaced with fresh normoglycemic culture medium, and cells were returned to standard culture conditions (37 ${}^{\circ }$ C, 5% CO ${}_{2}$ ) for 4 h of reoxygenation. To assess autophagic flux, cells were treated with chloroquine (25 μ M; C6628, Sigma-Aldrich). Chloroquine was added 30 min before the onset of anoxia and maintained throughout both the anoxic and reoxygenation phases to inhibit lysosomal degradation, thereby allowing the evaluation of autophagic flux through the accumulation of LC3B-II and p62 36. At the end of the reoxygenation period, cells were harvested for subsequent protein analysis.

### DNA fragmentation

H9c2 cells were seeded on coverslips in 6-well plates at 3 × 10 ${}^{4}$ cells/well, differentiated as above and subjected to A/R. At the end of the experiments, the samples were immediately processed for DNA fragmentation analysis. TUNEL was performed using the ‘TUNEL DeadEnd™ Fluorimetric’ (Promega, Italy) commercial kit. Briefly, cells were washed in PBS, fixed in 4% paraformaldehyde for 15 min and permeabilised with 0.1% Triton X-100 for 5 min. After incubation in equilibration buffer, the cells were treated with the TUNEL mix for 60 min in the dark at 37 ${}^{\circ }$ C. At the end of the incubation, the reaction was stopped, and the slides were washed three times with 0.1% Triton X-100 solution in PBS containing 5 mg/ml BSA (bovine serum albumin). Cells were then treated for 5 min with DAPI solution and mounted with Fluoromount™ (F4680, Sigma-Aldrich). Imaging analysis was performed using a Leica AF6500 Integrated System for Imaging and Analysis (Leica Microsystems, Wetzlar, Germany) equipped with the LAS software (LAS X Life Science). DAPI stains apoptotic and nonapoptotic cells blue while fluorescein-12-dUTP stains only the nuclei of apoptotic cells green. Nine non-overlapping fields per condition were acquired from three independent experiments. To calculate the percentage of apoptotic nuclei, we counted the number of TUNEL staining green fluorescence nuclei divided by the total DAPI positive blue fluorescence nuclei, and × 100. Positive control was performed by adding 3% H ${}_{2}$ O ${}_{2}$ for 4 h.

### RNA isolation and quantitative real-time PCR (qPCR)

H9c2 cells were seeded in a 6-well plate with a density of 3 × 10 ${}^{4}$ cells/well for controls and 6 × 10 ${}^{4}$ cells/well for differentiation. Total RNA was isolated, as previously indicated 37, using Direct-zol RNA MicroPrep Kits (C1003, Zymo Research, Irvine, CA, USA) following the manufacturer’s instructions. Total RNA was determined spectrophotometrically by Nanodrop (Thermo Fisher Scientific, Waltham, MA, USA). 250 ng of total RNA was used to synthesize cDNA using a high-capacity kit by iScript Adv cDNA kit for RT-qPCR (Bio-Rad) following the manufacturer’s recommendations. The reaction of qPCR was performed on a StepOne™ Real-Time PCR System instrument (Applied Biosystems Group, Foster City, CA) with SsoAdvanced Universal SYBR® Green Supermix (Bio-Rad) according to the manufacturer’s instructions. Primers for CD36 (qRnoCED0007736, Bio-Rad) and TnTc (qRnoCID0007317, Bio-Rad) were used to determine the gene expression levels. GAPDH (qRnoCID0057018, Bio-Rad) was used as a housekeeping gene for normalization. The 2 ${}^{\mathrm{-\Delta \Delta Ct}}$ method was used to calculate the expression levels of these genes (Livak KJ, Schmittgen TD).

### Western Blotting analysis

H9c2 cells were seeded in a 6-well plate at 3 × 10 ${}^{4}$ cells/well (controls) and 6 × 10 ${}^{4}$ cells/well (differentiation). After differentiation, cells were lysed in RIPA buffer (50 mM Tris-HCl, pH 7.5; 150 mM NaCl; 1% (vol/vol) Triton X-100; 1% (wt/vol) sodium deoxycholate; 0.1% (wt/vol) SDS; 1 mM Na2CO3; 25 mM NaF; and protease inhibitor cocktail (P8340, Sigma-Aldrich). 50 ug of total proteins were resolved on 10 or 15% SDS-PAGE under reducing conditions and electro-transferred onto nitrocellulose membranes. Membranes were blocked with 5% (wt/vol) non-fat dry milk dissolved in TBST (20 mM Tris, 150 mM NaCl, 0.1% Tween-20, pH 7.6) for 1 h at room temperature, incubated overnight at 4 ${}^{\circ }$ C with primary antibodies and then with HRP-conjugated secondary antibodies for 1 h at room temperature. Signals were detected using ChemiDoc (Bio-Rad).

### Immunofluorescence

H9c2 cells were seeded on coverslips in 6-well plates at 1 × 10 ${}^{4}$ cells/well (controls) and 3 × 10 ${}^{4}$ cells/well (differentiation). After 6 days, cells were washed, fixed with 4% formaldehyde for 10 minutes, and permeabilized with 0.2% Triton-X100 for 5 minutes when required. Slides were blocked with 5% BSA (A9418, Sigma-Aldrich) for 30 minutes, incubated overnight at 4 ${}^{\circ }$ C with primary antibodies, and subsequently with Alexa Fluor® 555- or 488-conjugated goat anti-rabbit IgG for 1 h at room temperature. Nine non-overlapping fields per condition were acquired from three independent experiments using identical microscope settings on a Zeiss LSM700 confocal microscope (Zeiss, Oberkochen, Germany), and fluorescence intensity was analyzed using ImageJ software (NIH Image; https://imagej.nih.gov/ij/).

### Antibodies used in the research

Anti-GLUT1 (PA5-16793, rabbit, 1:500) was obtained from Thermo Fisher Scientific® (Waltham, MA, USA); anti-LC3B (#2775, rabbit, 1:1000), anti-TnTc (#5593, rabbit, 1:1000), anti-Caspase3 (#9662, rabbit, 1:500), anti-cleaved Caspase3 (#9661, rabbit, 1:500), anti-PARP (#9542, rabbit, 1:1000), anti SQSTM1/p62 (#5114, rabbit, 1:1000) and anti-CD36 (#28109, rabbit, 1:1000) were acquired from Cell Signaling Technology (Boston, MA USA). Anti- β -actin (A2228, mouse, 1:1000) was purchased from Sigma-Aldrich (St. Louis, MO, USA). Anti-CPT1 (sc393070, mouse, 1:500) and anti-CD36 (sc-7309, rabbit, 1:500) were obtained from Santa Cruz Biotechnology (Dallas, TX, USA). Anti-mouse and rabbit HRP-conjugated secondary antibodies (170–6516 and 172-1019, respectively) were obtained from Bio-Rad (Hercules, CA, USA) and used at a concentration of 1:6000. Alexa Fluor® 555 and 488 goat anti-rabbit IgG (A-21428 and A-11008, respectively, 1:1000) were purchased from Thermo Fisher Scientific® (Waltham, MA, USA).

### Statistical analysis

Data derived from three independent experiments (n=3) performed on separate days using different cell passages between 10 and 20. Technical replicates were included where specified for plate-based assays. For imaging analyses, multiple non-overlapping fields were acquired per condition using identical acquisition settings. These technical measurements were first averaged within each independent experiment, and the resulting mean value was used as a single biological replicate for statistical analysis. Densitometry, fluorescence intensity and roundness index analysis were performed with ImageJ (NIH Image; https://imagej.nih.gov/ij/). No datasets were excluded from the analyses. The data obtained were analyzed with GraphPad Prism 8.3.0 (GraphPad Software, Inc., San Diego, CA). All data sets from the experiments were first analyzed for normality using the Shapiro–Wilk or Kolmogorov–Smirnov test. Comparisons between two groups were performed using an unpaired two-tailed Student’s t-test. For comparisons among three groups, one-way ANOVA followed by Dunnett’s multiple comparisons test (vs normoglycemic control) was used. Data are represented as mean ± standard error of the mean (SEM) of three separate experiments. P-values less than or equal to 0.05 were taken as significant.

## AUTHOR CONTRIBUTIONS

F.I. and L.E. conceived and designed research; S.P., A.I., L.C., C.D.G. and R.R. performed experiments; S.P., C.D.G. and A.I. analyzed data; F.I., L.E. and S.P. interpreted results of experiments; S.P. and A.I. prepared figures; S.P., F.I., and L.E. drafted manuscript; F.I., L.E., A.L. and S.P. edited and revised manuscript; F.I. approved final version of manuscript.

## SUPPLEMENTAL MATERIAL

All supplemental data for this article are available online at http://www.cell-stress.com/researcharticles/2026a-passaponti-cell-stress/. 

## CONFLICT OF INTEREST

The authors declare no conflict of interest.
