# Supplementary material for: Glycemia reprograms substrate transport and shapes divergent stress responses in differentiating H9c2 cells
Source: Cell Stress. 2026 Oct 5;10:74–87. doi: 10.15698/cst2026.10.321 (PMC13659450; doi:10.15698/cst2026.10.321)

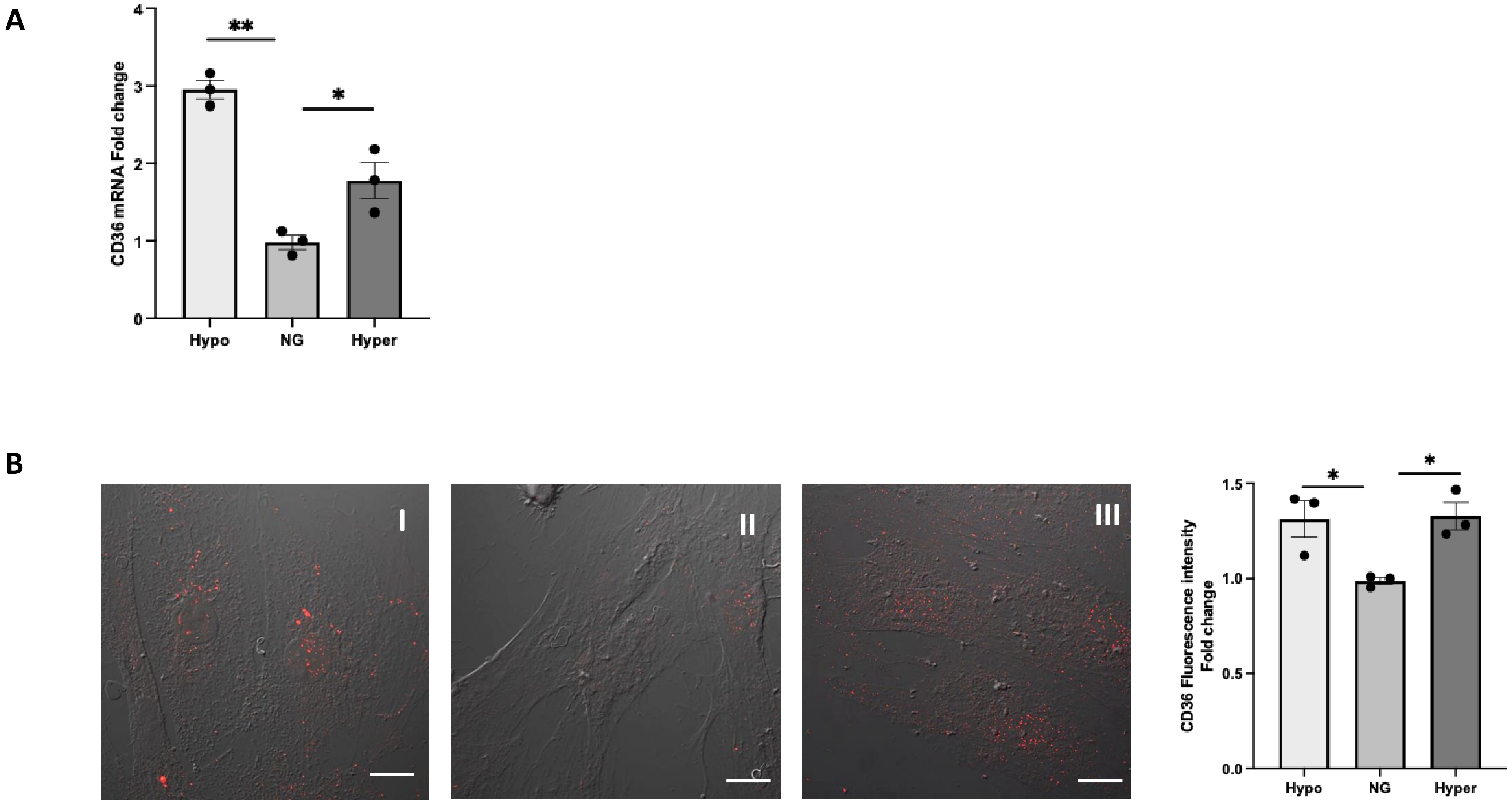

Supplementary Figure S1. CD36 gene and protein expression in H9c2 cells differentiated under different glucose conditions. (A) Relative CD36 mRNA expression in H9c2 cells differentiated under hypoglycemic (Hypo), normoglycemic (NG), and hyperglycemic (Hyper) conditions. CD36 transcript levels were significantly increased in both hypoglycemic and hyperglycemic cells compared with normoglycemic controls (*p<0.05, **p<0.01). (B) Representative confocal images of cell-surface CD36 immunofluorescence in H9c2 cells differentiated under hypo(I), normo(II), and hyper (III) conditions. CD36 is shown in red. Quantification of fluorescence intensity demonstrated a significant increase in CD36 expression in both hypo and hyper cells compared with normo (*p<0.05). Scale bar = 15 µm. Data are presented as the mean ± SEM of three independent experiments (N = 3). Statistical analysis was performed using one-way ANOVA followed by Dunnett’s multiple comparisons test.

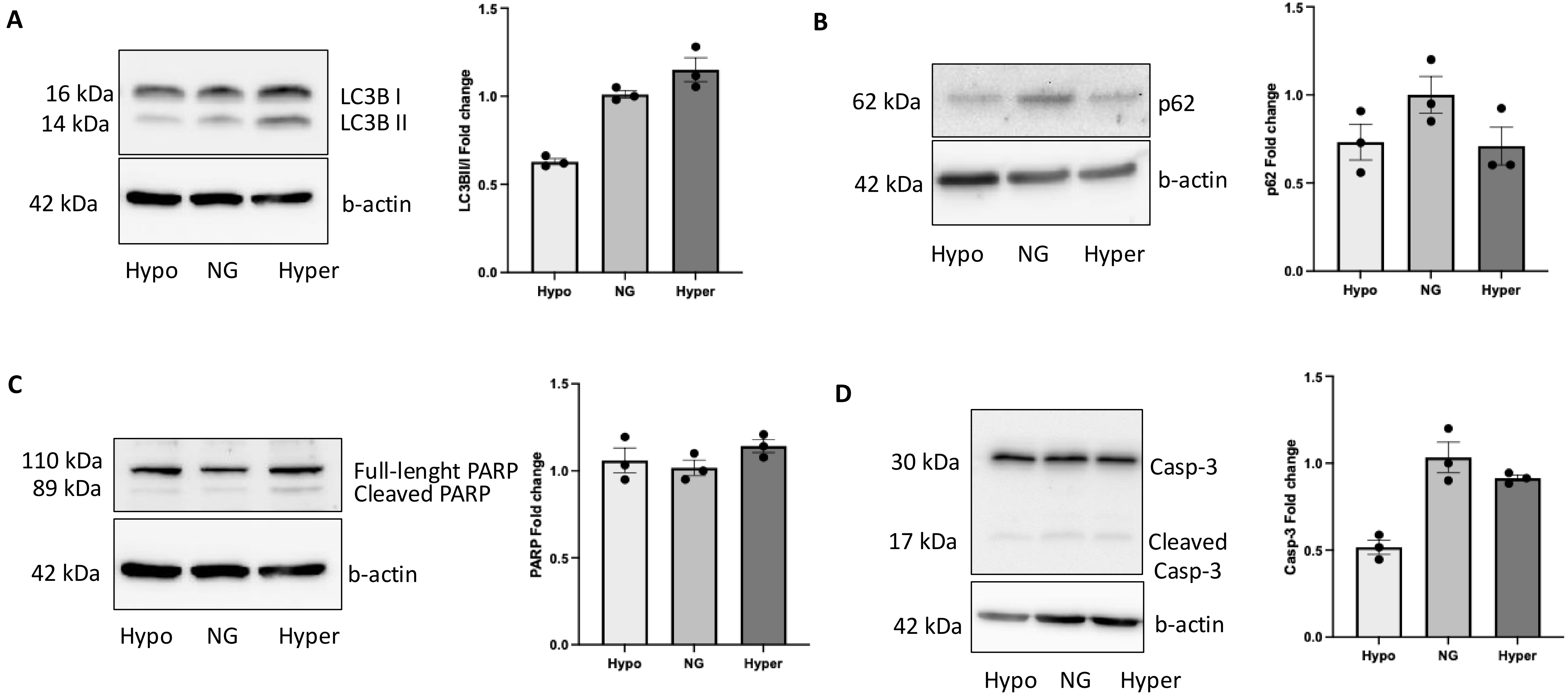

Supplementary figure S2. Basal autophagic and apoptotic status under the different glycemic conditions in differentiated H9c2 cells. Representative Western blot and densitometric analysis of the LC3B-II/LC3B-I ratio (A), p62 protein expression (B), the Cleaved PARP/Full lenght PARP ratio (C) and the Cleaved caspase 3/Total caspase 3 ratio (D) in H9c2 cells differentiated under hypoglycemic (Hypo), normoglycemic (NG), or hyperglycemic (Hyper) conditions before anoxia/reoxygenation. Data are expressed as fold change relative to each NG, and β-actin served as a loading control. Values represent mean ± SEM of three independent experiments (N=3). Statistical significance was determined by one-way ANOVA followed by Dunnett's multiple comparisons test.

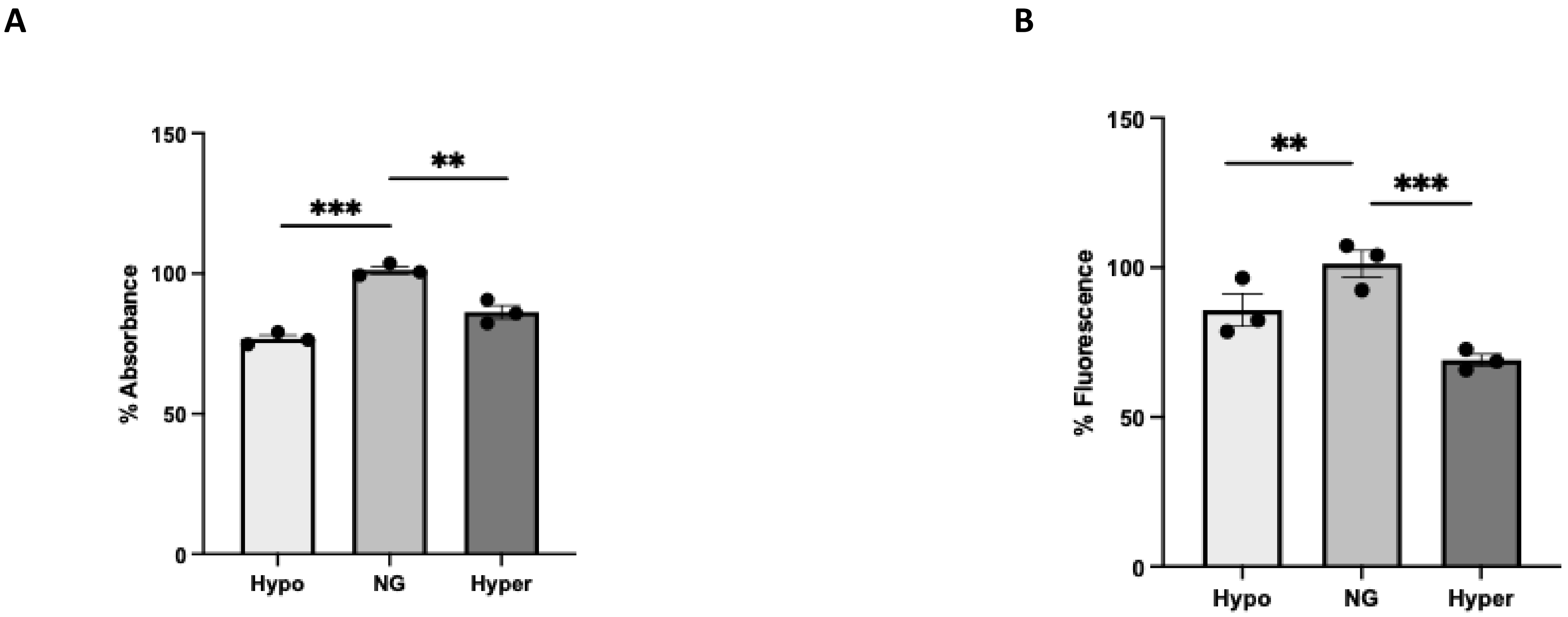

Supplementary figure S3. H9c2 cells' viability after A/R. Cell viability was assessed by MTT (A) and SRB (B) assays after the A/R protocol. Data are presented as the mean ± SEM of three independent experiments (N = 3). Statistical analysis was performed using one-way ANOVA followed by Dunnett's multiple comparisons test (**$p<0.01$, ***$p < 0.001$).

RAW BLOTS

Figure 1-C

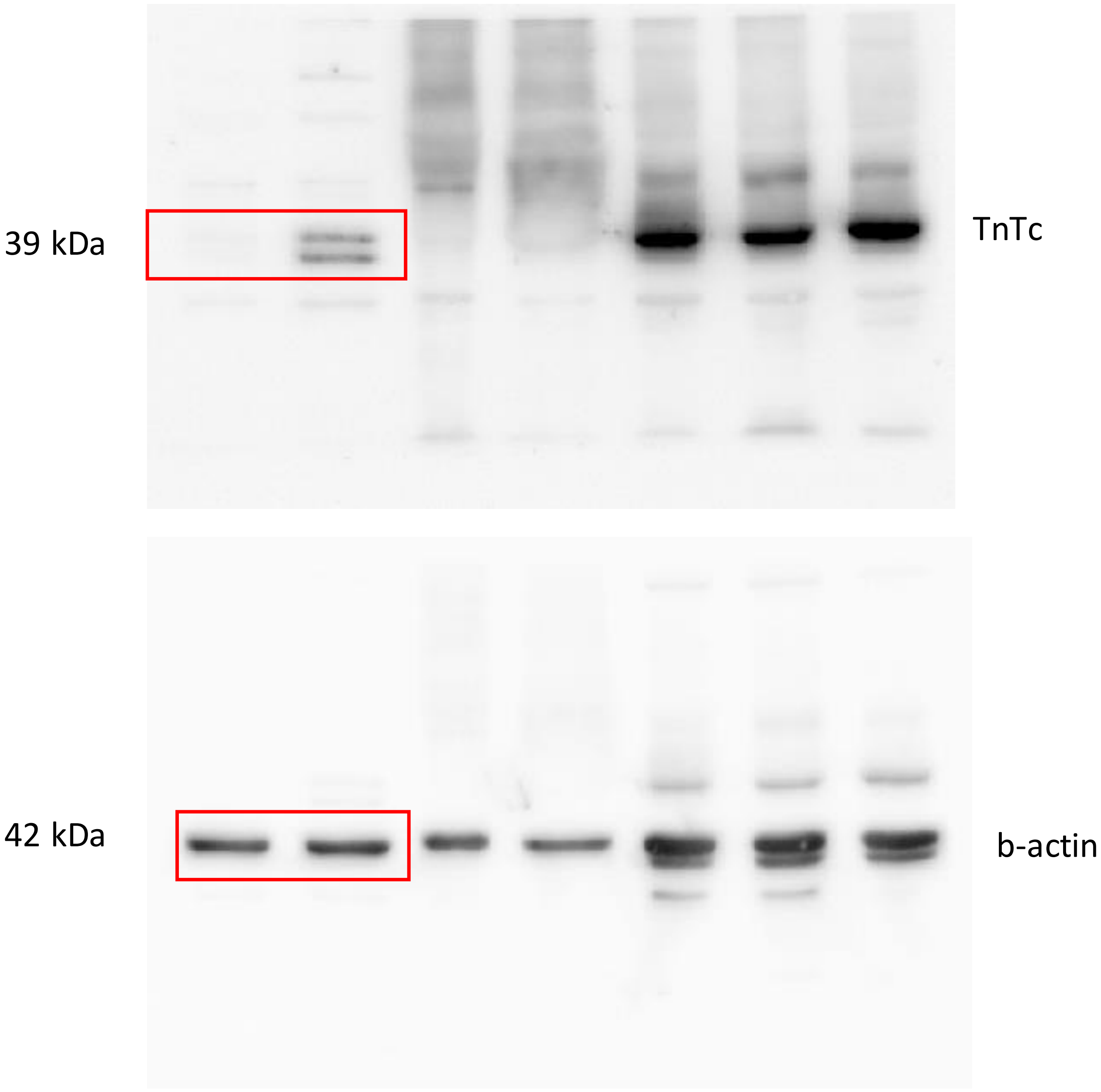

Figure 3-A

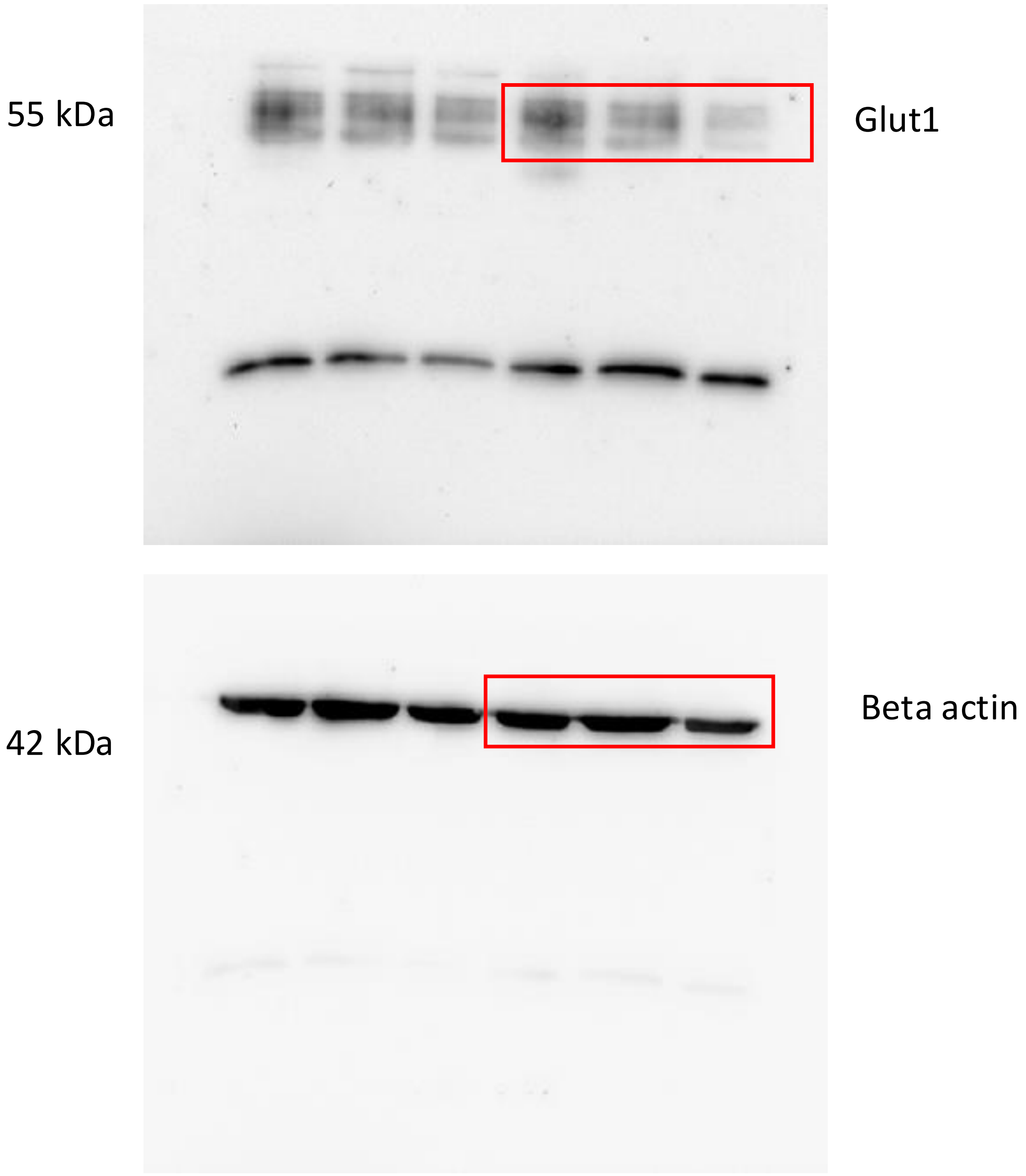

Figure 4-A

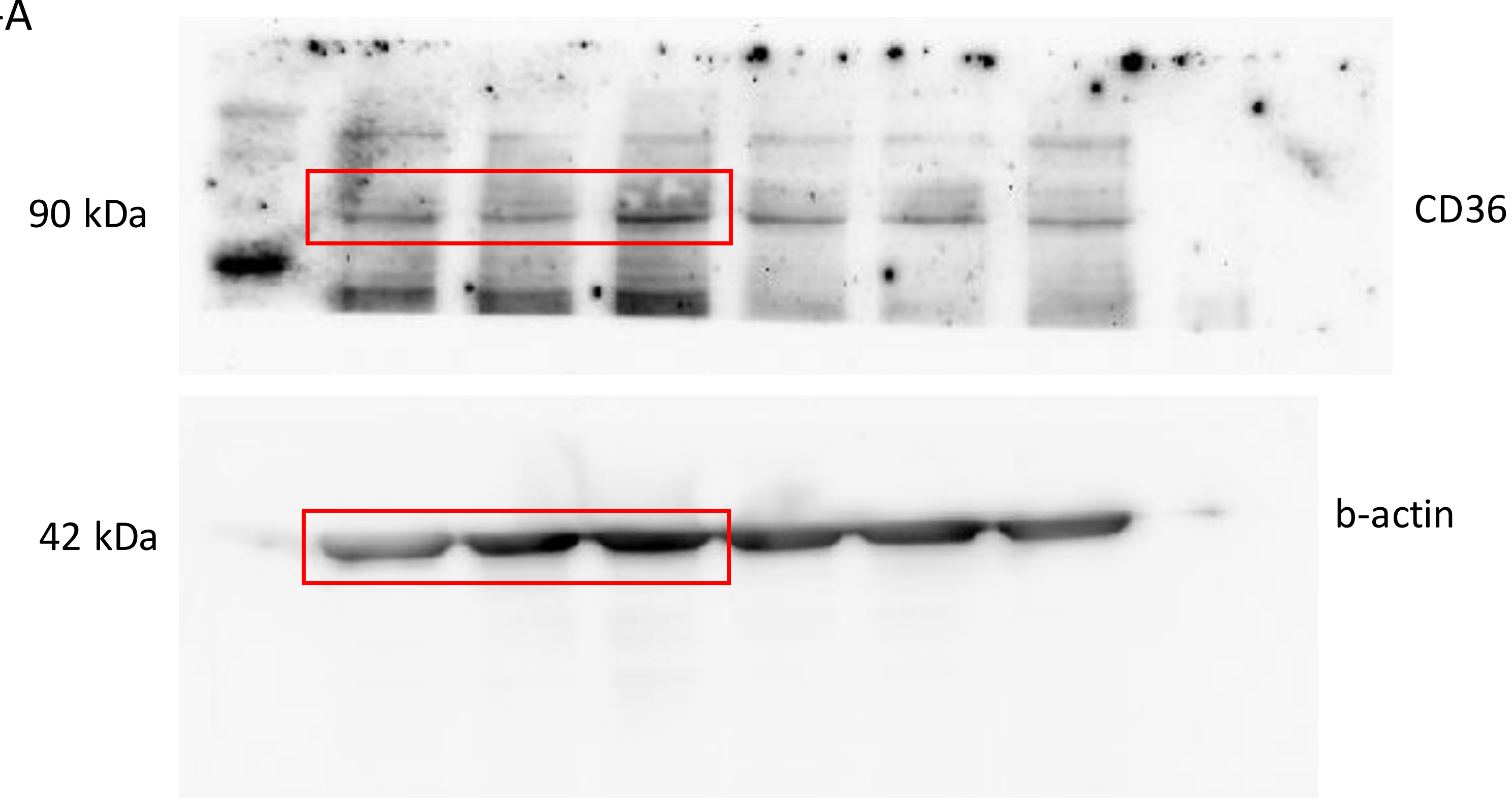

Figure 4-B

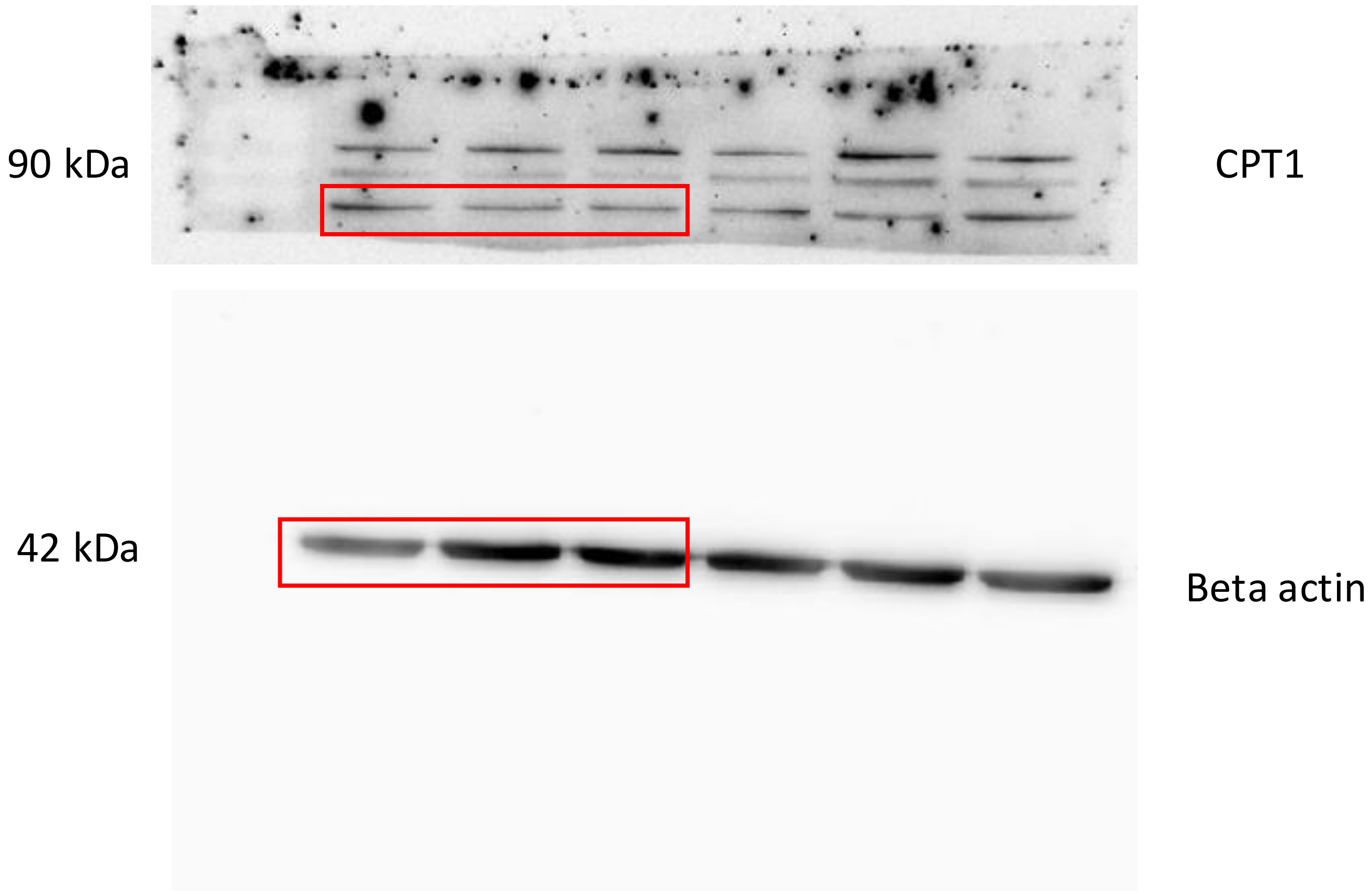

Figure 5-A,B

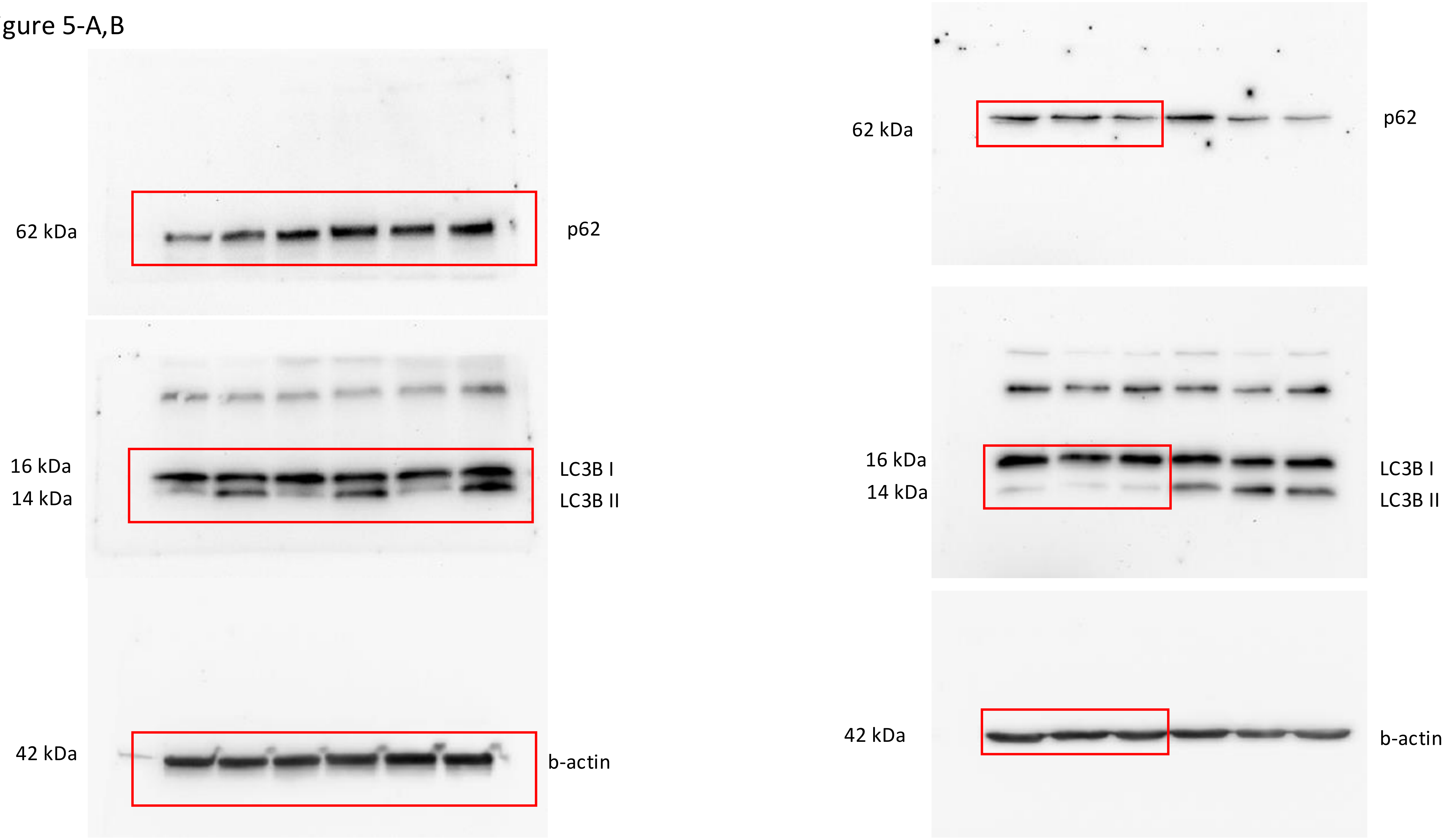
62 kDa
p62
16 kDa
14 kDa
LC3B I
LC3B II
42 kDa
b-actin
62 kDa
p62
16 kDa
14 kDa
LC3B I
LC3B II
42 kDa
b-actin

Figure 6-A

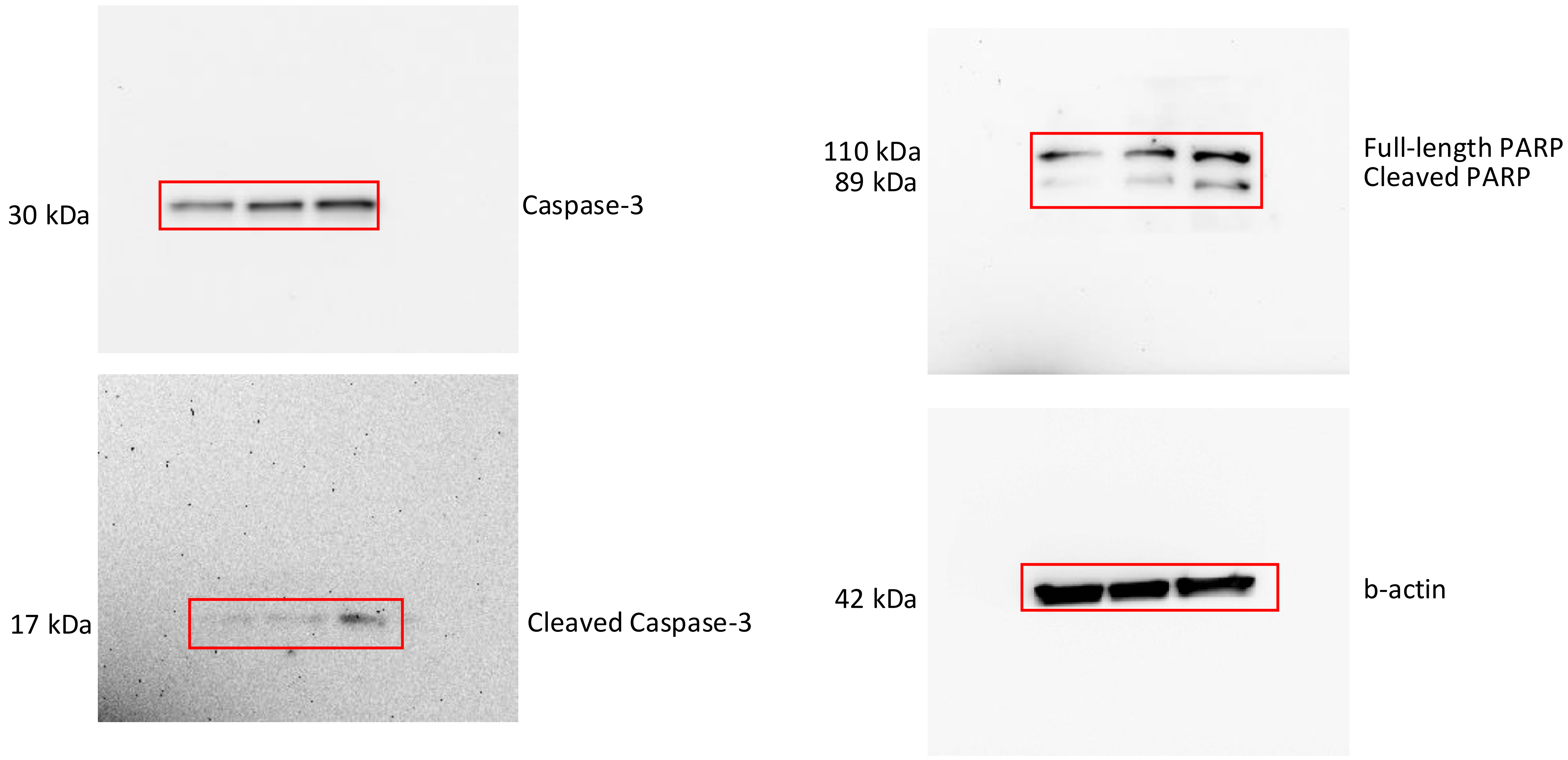

30 kDa
Caspase-3
17 kDa
Cleaved Caspase-3
110 kDa
89 kDa
Full-length PARP
Cleaved PARP
42 kDa
b-actin

Supplementary Figure S2

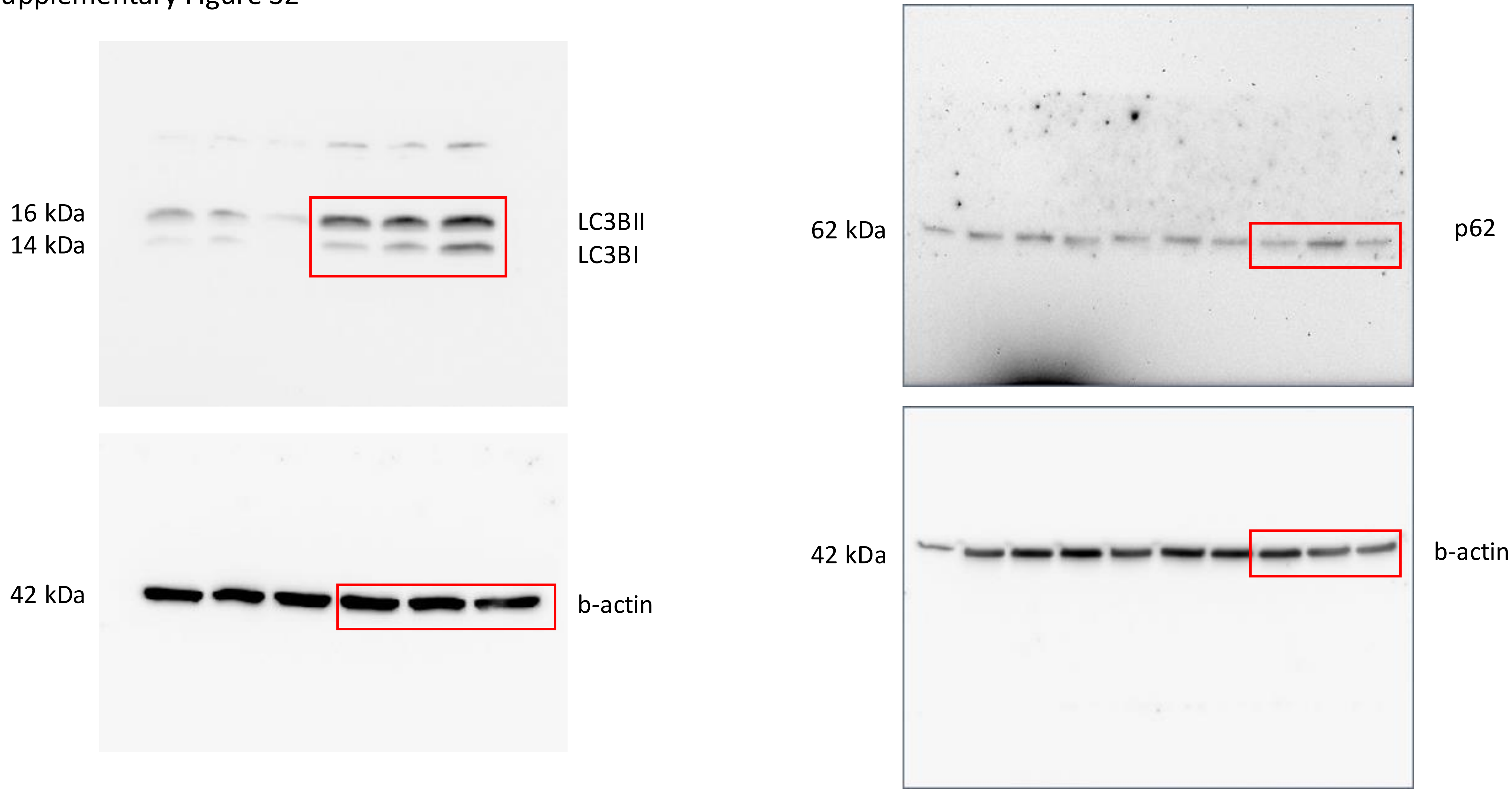

Supplementary Figure S2

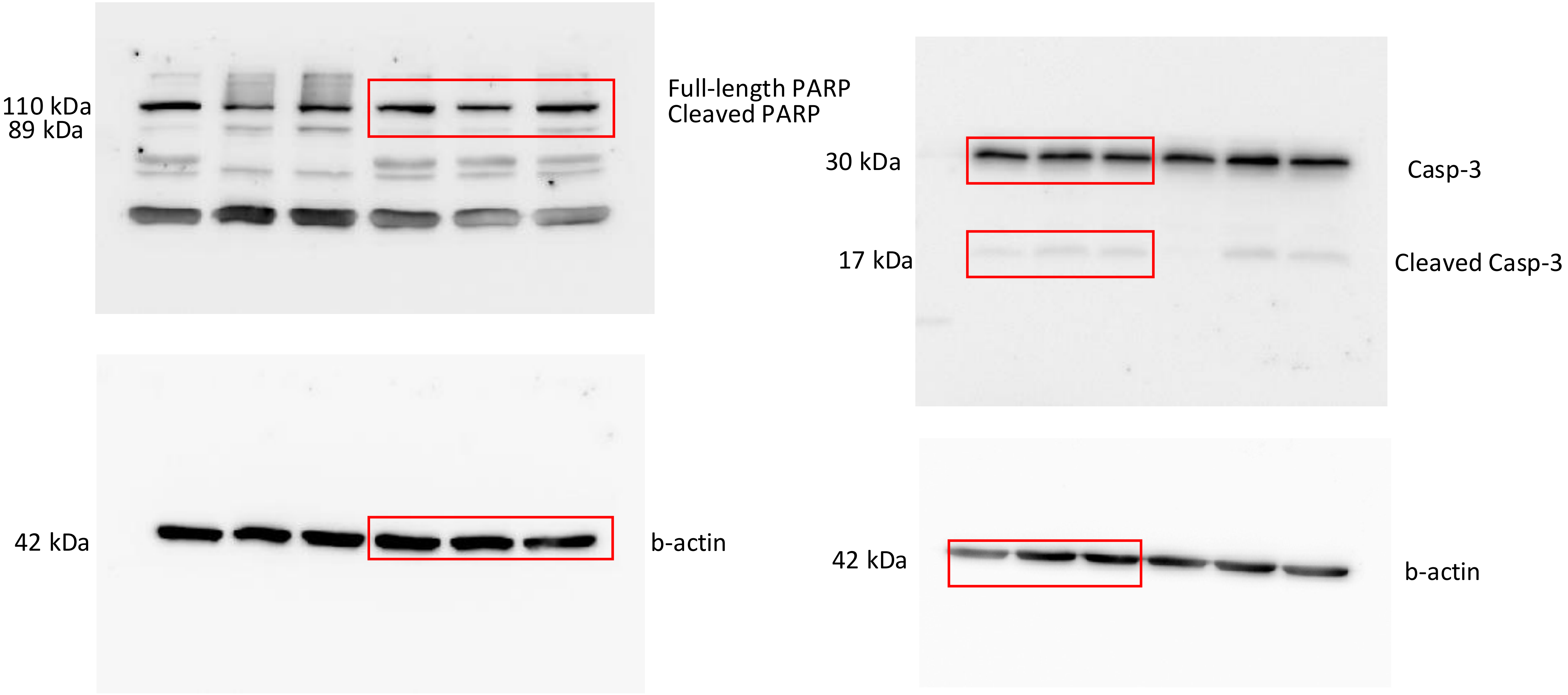

Supplement: Supplementary file 1 [file ces-10-074-s01.pdf]
